# Grapevine rootstocks shape underground bacterial microbiome and networking but not potential functionality

**DOI:** 10.1186/s40168-017-0391-2

**Published:** 2018-01-03

**Authors:** Ramona Marasco, Eleonora Rolli, Marco Fusi, Grégoire Michoud, Daniele Daffonchio

**Affiliations:** 1 King Abdullah University of Science and Technology (KAUST), Biological and Environmental Sciences and Engineering Division (BESE), Thuwal, 23955-6900 Saudi Arabia; 2 Department of Food Environmental and Nutritional Sciences, Università degli Studi di Milano, 20133 Milano, Italy

**Keywords:** Grapevine, Bacterial recruitment, Rootstock selection, Microbial ecology, Co-occurrence network, Plant growth-promoting bacteria, Microbiome, Rhizosphere, Endosphere

## Abstract

**Background:**

The plant compartments of *Vitis vinifera*, including the rhizosphere, rhizoplane, root endosphere, phyllosphere and carposphere, provide unique niches that drive specific bacterial microbiome associations. The majority of phyllosphere endophytes originate from the soil and migrate up to the aerial compartments through the root endosphere. Thus, the soil and root endosphere partially define the aerial endosphere in the leaves and berries, contributing to the *terroir* of the fruit. However, *V. vinifera* cultivars are invariably grafted onto the rootstocks of other *Vitis* species and hybrids. It has been hypothesized that the plant species determines the microbiome of the root endosphere and, as a consequence, the aerial endosphere. In this work, we test the first part of this hypothesis. We investigate whether different rootstocks influence the bacteria selected from the surrounding soil, affecting the bacterial diversity and potential functionality of the rhizosphere and root endosphere.

**Methods:**

Bacterial microbiomes from both the root tissues and the rhizosphere of Barbera cultivars, both ungrafted and grafted on four different rootstocks, cultivated in the same soil from the same vineyard, were characterized by 16S rRNA high-throughput sequencing. To assess the influence of the root genotype on the bacterial communities’ recruitment in the root system, (i) the phylogenetic diversity coupled with the predicted functional profiles and (ii) the co-occurrence bacterial networks were determined. Cultivation-dependent approaches were used to reveal the plant-growth promoting (PGP) potential associated with the grafted and ungrafted root systems.

**Results:**

Richness, diversity and bacterial community networking in the root compartments were significantly influenced by the rootstocks. Complementary to a shared bacterial microbiome, different subsets of soil bacteria, including those endowed with PGP traits, were selected by the root system compartments of different rootstocks. The interaction between the root compartments and the rootstock exerted a unique selective pressure that enhanced niche differentiation, but rootstock-specific bacterial communities were still recruited with conserved PGP traits.

**Conclusion:**

While the rootstock significantly influences the taxonomy, structure and network properties of the bacterial community in grapevine roots, a homeostatic effect on the distribution of the predicted and potential functional PGP traits was found.

**Electronic supplementary material:**

The online version of this article (10.1186/s40168-017-0391-2) contains supplementary material, which is available to authorized users.

## Background

Plant-specific microbiomes play an indisputable role in supporting plant health and adaptation to changing environmental conditions. These microbial communities act as a highly diversified, external secondary genome for the host plant and supply key ecological functionalities that contribute to increased plant fitness [1]. The final quality of plant products, such as fruit and other derived products, is dependent not only on the plant cultivar and cultivation practices but also on an ensemble of poorly characterized factors, grouped under the broad term *terroir* [2, 3]. In grapevines, the microbiomes associated with the phyllosphere and the fruits (carposphere) in particular were recently shown to present biogeographic-specific traits that further define the *terroir* properties [2–4]. High-throughput sequencing analysis demonstrated that the non-random ‘microbial *terroir*’ was a determining factor in regional grape must characteristics, showing that microbial vineyard inhabitants play a critical role in determining fruit quality [2, 5, 6].

Interestingly, the majority of the microbial taxa found in the aboveground grapevine tissues originated in the soil, indicating that the roots act as the primary reservoir of bacterial grapevine colonizers [7, 8]. In particular, the phyllosphere endophytes migrate to the aerial tissue from the root endosphere after they are recruited by the root tissues from the soil, which has a determinant role as a microbial supplier for the aerial endosphere [7–9]. It can be argued that the plant species and genotype also play important roles in selecting the plant-specific endophytes from soil [8].

Since the nineteenth century, *Vitis vinifera* varieties have been cultivated as scions and grafted onto the rootstocks of other *Vitis* species and hybrids to prevent vineyards from succumbing to *Phylloxera* pests. Grafting *Phylloxera*-immune rootstock is a global practice, and the development of new rootstock genotypes is an important aspect of modern viticulture [10]. The rootstock affects scion development by influencing the reproductive performance, vigour, biomass accumulation and distribution in the plant, phenology and fruit yield [11–13]. Moreover, the rootstock influences plant resistance to soil-borne pests [14], climate, or adverse environmental and soil conditions, such as drought [15], salinity [16], limestone content [17] and poor mineral nutrition [18]. Different rootstocks often co-occur in the same vineyard to maximize plant resilience to adverse growth conditions.

Although it is widely known that the rootstock genotype and the grape-associated microbiota affect the grapevine physiology, studies unveiling the structure of the bacterial assemblages associated with different graft combinations are lacking [13]. Here, we hypothesize that the rootstock genotype plays a fundamental role in influencing the recruitment and structure of the bacterial microbiome associated with the rhizosphere and root endosphere. Considering that the root endosphere mediates the passage of endophytes to the aerial endosphere and carposphere [7–9], understanding the effect of the rootstock type on the microbiomes in the root endosphere is a necessary step towards understanding the factors that determine the microbial *terroir* of grapevines. To address effects of the rootstock type on the selective recruitment of bacterial communities in the rhizosphere and root endosphere, we compared ungrafted and grafted plants of the cultivar Barbera, all cultivated in the same soil at a single vineyard. We investigated the bacterial diversity in five different root systems with 16S rRNA gene high-throughput sequencing and inferred the predicted functionality and interactions in the root tissues and rhizosphere. Furthermore, we used a cultivation-dependent approach to test the plant growth-promoting (PGP) functionality of the cultivable bacterial strains detected by high-throughput sequencing.

## Results

### Richness and diversity of bacterial communities associated with the root systems of ungrafted grapevine or grapevine grafted on different rootstocks

**Fig. 1:**
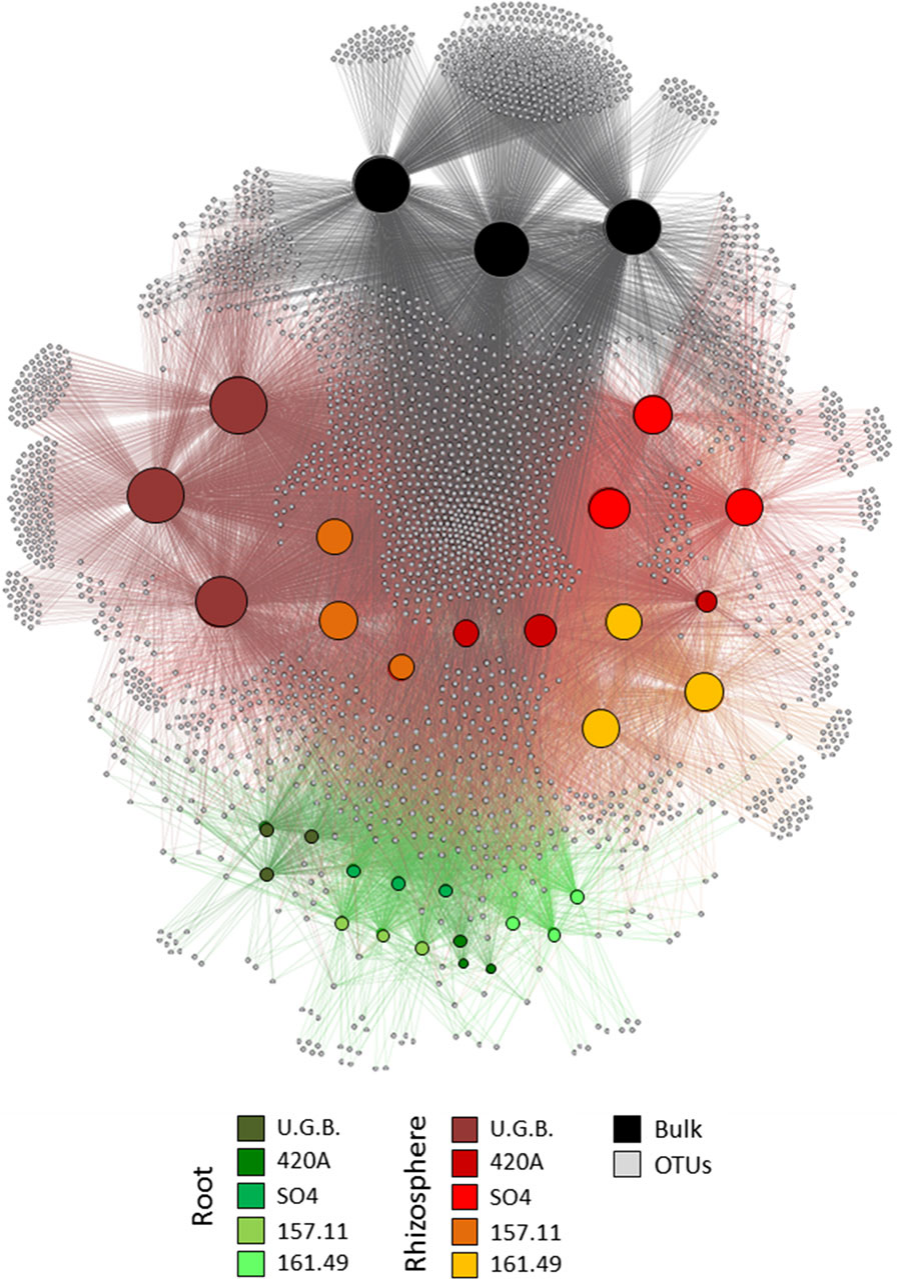
Bipartite network analysis of grape root-system bacterial communities. Bipartite networks representing sample/OTU interactions. In both networks, edge visibility (line width and opacity) was enhanced by weights to highlight the most relevant connections. Sample nodes (circles) are shown according to grapevine rootstock and fraction; OTU nodes are grey, with the edges connecting sample nodes to OTU nodes coloured by sample type (rootstock/fraction of origin). Black = bulk, Red nuance = rhizosphere, Green nuance = root. Each node size is proportional to its degree of connection

**Fig. 2:**
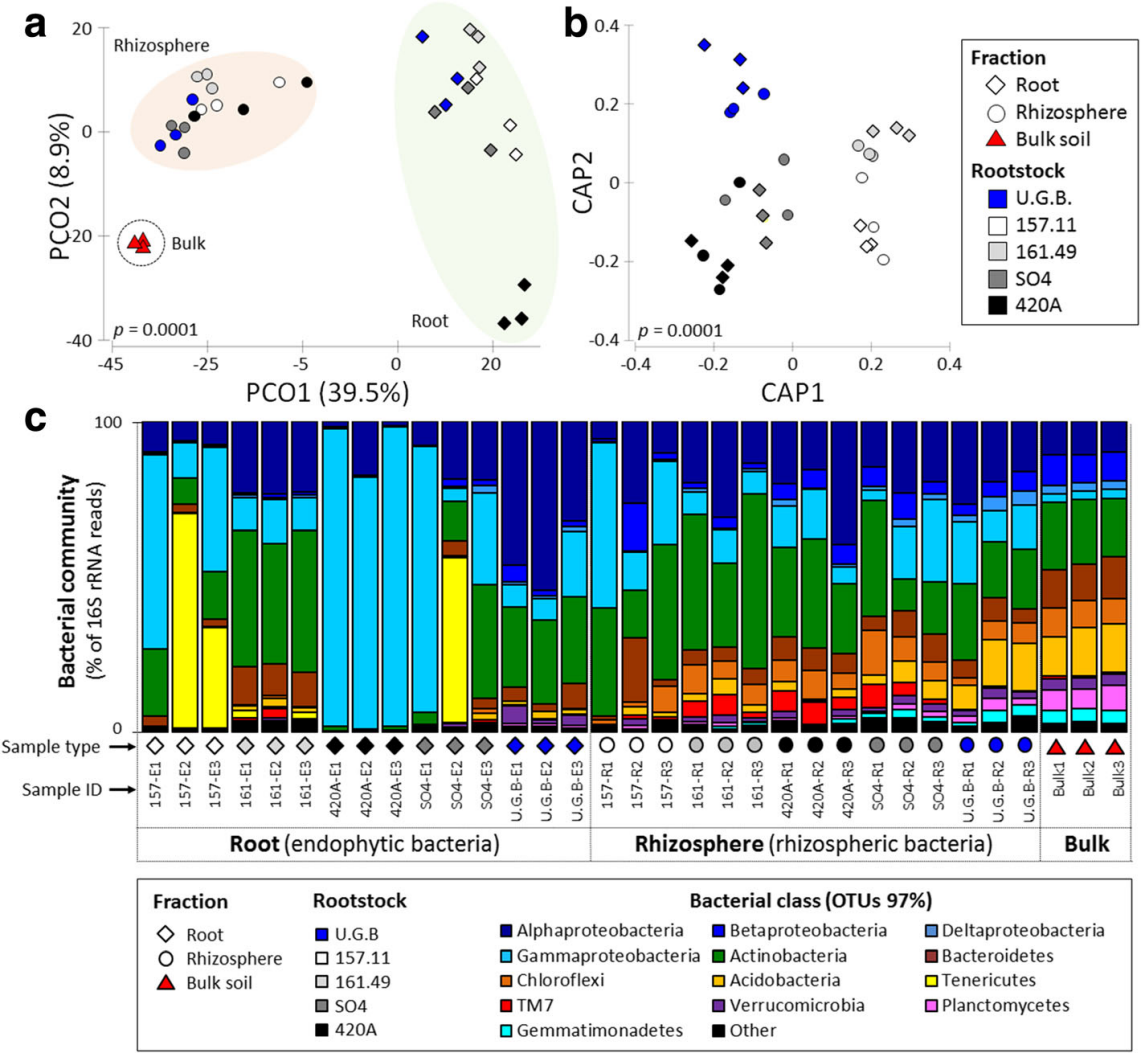
Comparison of microbial communities in samples from different rootstock root-system compartments (root tissues and rhizospheres). **a** Principal coordinates analysis (PCoA) for root, rhizosphere and bulk samples. **b** Constrained analysis of principal coordinates (CAP) was generated from the same OTU table by selecting only those samples influenced by their location within the root system (root tissue or rhizosphere) and constrained to the rootstock grouping factor. Pairwise comparisons using permutational MANOVAs on a distance matrix are shown in Additional file 1: Table S4. **c** Relative abundance of different bacterial classes in the root tissues and rhizospheres of rootstocks representing OTUs showing more than 1% relative abundance of all reads and present in at least 2/3 of replicates. Classes representing less than 1% of the total reads are grouped in ‘Other’

The bacterial diversity of the Barbera cultivar, ungrafted (U.G.B., *V. vinifera*) and grafted on four different rootstocks (*V. riparia* × *V. berlandieri*: SO4, 420A, 161.49 and 157.11; Additional file 1: Table S1 and Additional file Rootstock characterization), was determined in the rhizosphere and root endosphere by Illumina MiSeq sequencing of 16S rRNA gene (Additional file 1: Figure S1). A total of 2586 different operational taxonomic units (OTUs) were identified (163 ± 59, 918 ± 295 and 1533 ± 50 in root endosphere, rhizosphere and bulk soil, respectively, Additional file 1: Table S2) corresponding to a total of 552,094 paired-end reads (10,497 ± 7520, 21,704 ± 6168 and 23,024 ± 2251 in the root endosphere, rhizosphere and bulk soil, respectively, Additional file 1: Table S2). A bipartite network analysis showed that the bulk soil harbours the highest number of OTUs, which are shared in part with the plant-associated fractions, i.e. the root tissue and rhizosphere (Fig. 1). Among the soil OTUs (2537), 48% were shared between the rhizosphere and bulk soil samples, while only 10% were in common with the root tissues. Principal coordinate analysis (PCoA) revealed a strong clustering of bacteria at the OTU level (97% identity) according to the different root fractions (root tissues, rhizosphere and bulk soil; Fig. 2a and Additional file 1: Table S3A). The interaction of the two factors ‘Fraction’ and ‘Rootstock Type’ significantly affected beta-diversity (PERMANOVA: df = 4,29, *F* = 5.12, *p* = 0.001, Additional file 1: Table S4 and Additional file 1: Table S5A), explaining 11.6% of the observed variation (Additional file 1: Table S5B), in addition to the one explained by Fraction factor (46.4% of total variance) and the Rootstock factor (10% of the total variance, Additional file 1: Table S5B). The remaining part (32%) of the observed variance remain unresolved and cannot be explained considering only these two factors (Additional file 1: Table S5B). A fraction/rootstock-specific bacterial community was identified (Additional file 1: Figure S2A and B). Bacterial assemblage in the root tissues showed rootstock-specific endophytic bacterial communities (Additional file 1: Table S3B). Similarly in the rhizosphere, the rootstock influenced the bacterial assemblage, with only one pair of rootstocks (420A, SO4) that not differentiate significantly (Additional file 1: Table S3C).

The edge distribution and density in the bipartite network indicated the different distributions of OTUs among the factors ‘Fraction’ and ‘Rootstock Type’. Specific OTUs were connected to the five different rhizosphere samples, especially in the case of ungrafted plants (U.G.B., Additional file 1: Table S6). A similar trend, accounting for significantly less OTUs per sample, was found in the root tissues. A significant difference in the alpha diversity was found between the ‘Fraction’ and ‘Rootstock Type’ factors, with higher Shannon indices and OTU-Richness values in the bulk soil and rhizosphere than in the root tissues (Additional file 1: Table S2). The alpha diversity of the bacterial microbiomes associated with the different rootstocks was variable, with 420A showing significantly less diversity than the other rootstocks, both in the root and in the rhizosphere (Additional file 1: Table S2 and Additional file 1: Figure S3).

### Bacterial taxa distribution in the grape root system is significantly influenced by the fraction and the rootstock types

According to the taxonomic affiliations of the OTUs, the grapevine root system hosted 35 bacterial phyla (Additional file 1: Table S7A), 105 classes (99.7% sequences classified; Additional file 1: Table S7B), 149 orders (94.7% classified; Additional file 1: Table S7C), 184 families (83.9% sequences classified; Additional file 1: Table S7D) and 182 genera (44.2% sequences classified; Additional file 1: Table S7E). Both ungrafted and grafted Barbera grape root systems were dominated by *Proteobacteria* (53%: *Gammaproteobacteria* and *Alphaproteobacteria* 31 and 17%, respectively), *Actinobacteria* (24%), *Bacteroidetes* (5%), *Chloroflexi* (4%) and *Acidobacteria* (4%) phyla (Fig. 2c). While *Proteobacteria*, *Actinobacteria* and *Bacteroidetes* relative abundance did not show significant changes among root and rhizosphere fractions (false discovery rate, FDR: *p* > 0.05), *Chloroflexi* and *Acidobacteria* were significantly more abundant in the rhizosphere (FDR: *t* = 7.39, *p* = 0.003; *t* = 3.49, *p* = 0.003; respectively). Despite the distribution of *Proteobacteria* did not significantly differ among the two fractions, classes belonging to this phylum presented different trends. For instance, *Betaproteobacteria* (FDR: *t* = 3.83, *p* = 0.003) and *Gammaproteobacteria* (FDR: *t* = 2.39, *p* = 0.048) were differently distributed among the two fractions, while *Alphaproteobacteria* presented a similar (FDR: *t* = 0.245, *p* = 0.8) relative abundance across the grape root system.

Among the sequences identified at the genus level (Additional file 1: Table S7E and Additional file 1: Figure S4), the main genera in the root tissues were ‘*Candidatus* Phytoplasma’ (FDR: *t* = 1.85, *p* = 0.006) and *Pseudonocardia* (FDR: *t* = 2.78, *p* = 0.003), while *Pseudomonas* dominated both in the root and the rhizosphere fractions (FDR: *t* = 1.54, *p* = 0.26).

**Fig. 3:**
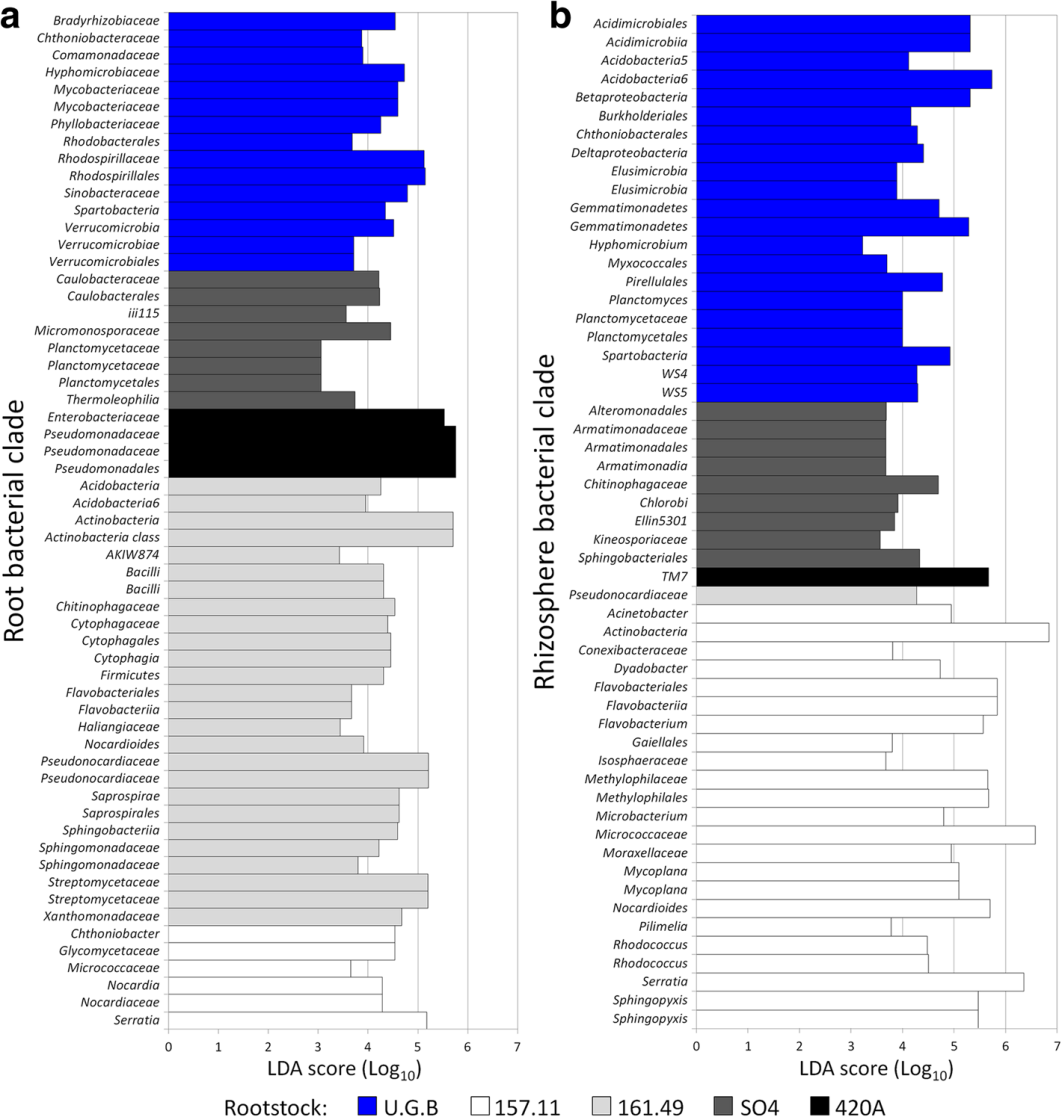
Peculiar clades among the bacterial communities associated with grafted and ungrafted grape root and rhizosphere. The bar charts report the taxonomic representation of statistically and biologically consistent differences between the bacterial communities associated with the different rootstocks, as determined by LEfSe analysis. Details regarding the LEfSe analyses are reported in Additional file 1: Table S6

Bacterial taxa distributions at phylum (Fig. 2c) were significantly influenced by the rootstock type in the root tissues (F_4,10_ = 5.5586; *p* = 0.001), but not in the rhizosphere (F_4,10_ = 1.9591). On the contrary, genera distribution (Additional file 1: Figure S4 and Additional file 1: Table S7E) revealed a rootstock effect in both root compartments (root: F_4,10_ = 3.6952, *p* = 0.014; rhizosphere: F_4,10_ = 2.6123, *p* = 0.105). The linear discriminant analysis effect size (LEfSe) detected 59 bacterial clades in the roots and 55 in the rhizospheres, which discriminated the bacterial communities between the different root genotypes (Fig. 3a, b, Additional file 1: Table S8). Highly specific distributions of the bacterial clades were observed in the root endosphere of the different root systems, with the dominance of *Actinobacteria* (83% of clades) in rootstock 157.11, *Proteobacteria* in both 420A (100%) and U.G.B. (53%), *Planctomycetes* (38%) in SO4 and *Bacteroidetes* (35%) in 161.49 (Fig. 3a and Additional file 1: Table S8A). In the rhizosphere, both rootstock 157.11 and U.G.B. showed higher numbers of differentially abundant clades (24 and 21, respectively) than the other three rootstocks (9, 1 and 1 in SO4, 420A and 161.49, respectively). In this compartment, new phyla were detected with increasing clade diversity associated with 157.11, SO4 and U.G.B.; 420A and 161.49 were dominated by one taxon each, TM7 and an *Actinobacteria* clade, respectively (Fig. 3b and Additional file 1: Table S8B).

The rootstock-pairs dissimilarity, due to phyla and genera contribution in root and rhizosphere fractions, was calculated by SIMPER (similarity percentages) analysis (Additional file 1: Table S9 and S10). Higher microbiome dissimilarity among rootstocks was revealed in the root tissues compared to the rhizosphere, both considering phyla (Additional file 1: Table S9A) and genera (Additional file 1: Table S9B) distribution. *Acidobacteria, Bacteroidetes* and *Chloroflexi* were the major phyla that contribute to differentiate the endophyte communities associated with the different rootstock types. In the rhizosphere, *Gammaproteobacteria*, *Betaproteobacteria* and *Actinobacteria* further contributed to the relative low dissimilarity (Additional file 1: Table S9A and Additional file 1: Table S10 A and B). Several genera determined the dissimilarities among rootstocks in both endosphere and rhizosphere fractions and some genera appeared to be root genotype biomarkers, such as *Novosphingobium* and *Streptomyces* in the rhizospheres of U.G.B. and SO4, respectively, and *Serratia* and ‘*C.* Phytoplasma’ in the root tissues of 157.11 and 161.49, respectively (Additional file 1: Table S9B and Additional file 1: Table S10 C and D).

### Rootstock-specific and shared bacterial assemblages

**Fig. 4:**
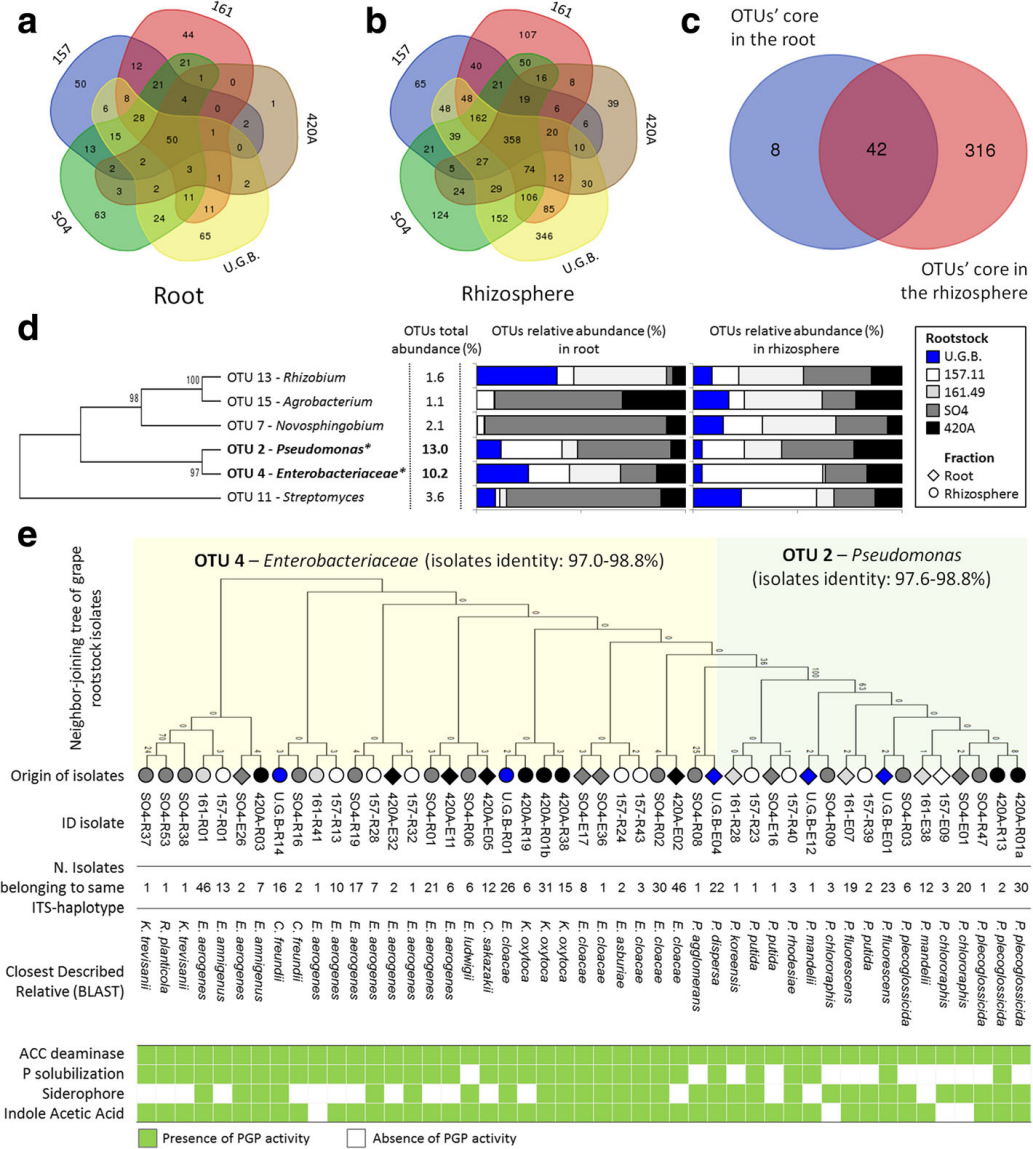
Grape rootstock shared microbiome and correlation with cultivable bacteria. **a**, **b** Venn diagrams showing the common and exclusive bacterial OTUs of the roots and compartments the selected rootstock. **c** Overlaps between the shared rhizosphere and root OTUs of all selected rootstocks. **d** Phylogenetic tree and distribution of the six most abundant (≥ 1%) shared OTUs among the root systems (root tissue and rhizosphere) of all selected rootstocks. **e** Phylogenetic tree of representative bacterial isolates having more than 97% similarity with the two most abundant shared OTUs, individuated by high-throughput sequencing data analyses. The identification and functional PGP traits of the selected isolates are shown

The root endosphere and rhizosphere compartments of grafted and ungrafted plants showed specific OTUs for each rootstock and a cluster of shared OTUs (Fig. 4a, b and Additional file 1: Table S6 A and B). The root tissues and the rhizosphere had cores with 50-shared (76% of the total root-tissue OTUs; Additional file 1: Table S11A) and 358-shared OTUs (74% of the total rhizosphere OTUs; Additional file 1: Table S11B), respectively. The shared root-tissue bacteria were primarily dominated by two OTUs phylogenetically affiliated with *Pseudomonas* (OTU 2, 29%) and *Enterobacteriaceae* (OTU 4, 24%), followed by OTUs belonging to *Actinomycetales* (9%) and *Rhizobiaceae* (4%; Additional file 1: Table S10A). The shared rhizosphere bacteria were *Actinomycetales* (23%), *Sphingomonadales* (7%) and *Rhizobiales* (7%), followed by *Pseudomonas, Enterobacteriaceae* and *Chloroflexi*, which together account for 15% of the bacterial community in the rhizosphere (Additional file 1: Table S11B). Specific OTUs associated with the rootstocks 420A, 157.11 and SO4 endospheres represented less than 2% of their bacterial communities, while those associated with 161.49 and U.G.B. represented 6.5 and 7.6% of their bacterial communities, respectively (Fig. 4a and Additional file 1: Table S12A). A similar trend was observed in the rhizosphere, where the U.G.B.-specific OTUs enriched 5% of the relative abundance (Additional file 1: Table S12B). A comparison between the shared rhizosphere OTUs (50) and the shared endosphere OTUs (358) revealed a total of 42 shared OTUs (mainly affiliated to *Gammaproteobacteria*, *Alphaproteobacteria* and *Actinobacteria*), counting for 42% of the sequenced relative abundance in the root system (Fig. 4c and Additional file 1: Table S11C). The top six shared OTUs represented 32% of the overall bacterial community and were taxonomically affiliated with *Rhizobium*, *Agrobacterium*, *Novosphingobium*, *Pseudomonas*, *Enterobacteriaceae* and *Streptomyces,* with a variable distribution in the root compartments of the different rootstocks (Fig. 4d).

### Co-occurrence networks in the bacterial communities of ungrafted and grafted Barbera root systems

**Fig. 5:**
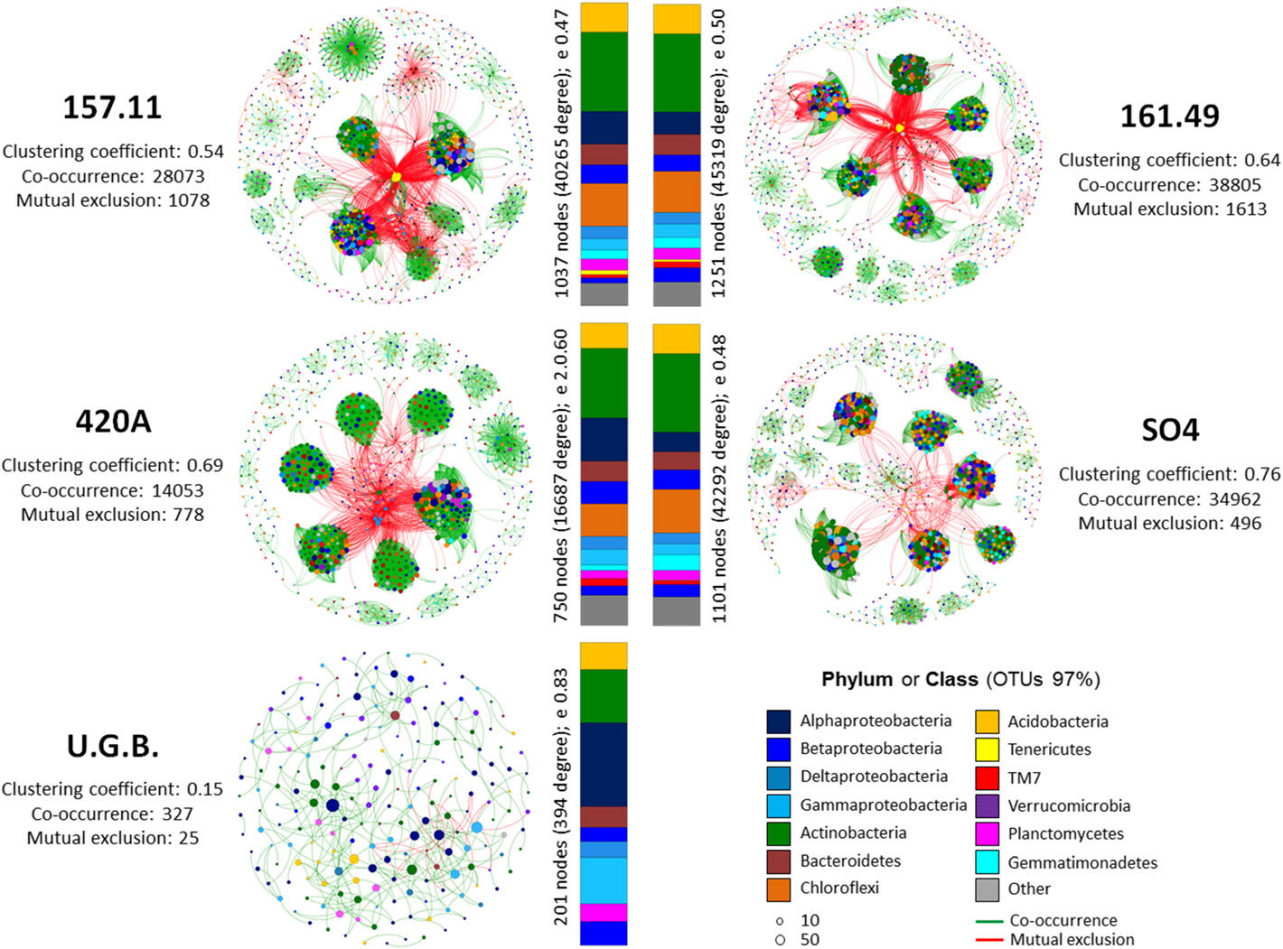
Significant co-occurrence network of bacterial communities associated with root systems of ungrafted and grafted grapevines. Interaction among the OTUs in root systems (root and rhizosphere fractions) of grafted (157.11, 161.49, 420A and SO4) and ungrafted (U.G.B.) grapevines. The nodes of each network are coloured according to phylum or class affiliation (97%) and sized according to degree of connection (Additional file 1: Table S13). The edges connecting the nodes are represented by green lines to indicate co-occurrence interactions and by red for mutualistic exclusions (Additional file 1: Table S14). Relative abundance of those nodes with degree of connection in the third percentile is reported at the phylum/class level in the bar chart for each grape type

An analysis of the co-occurrence bacterial networks within the root systems (root and rhizosphere) of grafted and ungrafted Barbera plants showed different connectivity patterns directly and differently influenced by the rootstock type (Fig. 5 and Additional file 1: Table S13). We recorded a significantly higher number of co-occurrence interactions than mutual exclusions in all of the networks. We recorded significantly more nodes, network clustering, interactions and network density in the grafted plants than in U.G.B. (Additional file 1: Table S13). These findings indicate a higher complexity of the bacterial networks in the root systems of the grafted grapes than in U.G.B. All of the grafted root systems featured compartmentalized bacterial modules, several of which (up to 66) were connected, and presented high numbers of connections (up to 179 per node; Additional file 1: Figure S5 and Additional file 1: Table S14). In contrast, the bacterial networks in the U.G.B. root systems presented only 16 modules and the connected taxa showed a similar degree of interactions (Additional file 1: Figure S5 and S6).

The taxonomy of the most connected nodes significantly differed between the grafted and ungrafted root systems (*p* = 0.008, Fig. 5, Table 1 and Additional file 1: Table S14). *Actinobacteria* mainly shaped the topology of the bacterial network in the grafted root system (up to 26% of the total degree of connection; Table 1), followed by *Chloroflexi* (up to 15%), *Alphaproteobacteria* (up to 14%) and *Acidobacteria* (up to 10%). In the U.G.B. root system, connectivity among the bacterial community was primarily driven by *Alphaproteobacteria* (28% of the total degree of connection; Table 1), followed by *Actinobacteria* (17%) and *Gammaproteobacteria* (15%). Several of the highly connected taxa, such as *Acidobacteria* (Ellin6075 and *Solibacteraceae*), *Actinobacteria* (*Gaiellaceae*, *Nocardiaceae* and *Microbacteriaceae*), *Alphaproteobacteria* (*Sphingomonadaceae* and *Rhodospirillaceae*) and *Gammaproteobacteria* (*Sinobacteraceae* and *Xanthomonadaceae*), appeared to be the key species driving the bacterial occurrence within the plant microbiome [19]. The most connected taxa established positive interactions (from 69 to 100% of the total interaction), while *Tenericutes* (representing 2.2% of the sequences in the dataset) established mainly negatives (from 79 to 100% of the total interaction), indicating exclusion-based interactions with many of the other community components (Tables 1, 2 and Additional file 1: Table S15). Phylogenetic analysis identified the *Tenericutes* OTU as closely related to ‘*Candidatus* Phytoplasma solani’ (Additional file 1: Figure S7), the causal agent responsible for bois noir disease [20]. Although the grafted Barbera roots (157.11, 161.49 and SO4 rootstocks) hosted relatively high numbers of *Tenericutes* in the root tissues (Fig. 2c and Table 2), the plants did not present any obvious symptoms of grapevine yellows disease.

**Table 1:** Degree of connection for each phylum/class in bacterial community networks of grapevine roots

Distribution of degree of connection	Grafted and ungrafted grape root system														
	157.11			161.49			SO4			420A			U.G.B.		
	Tot	(−)	(+)	Tot	(−)	(+)	Tot	(−)	(+)	Tot	(−)	(+)	Tot	**(−)**	**(+)**
	%	%	%	%	%	%	%	%	%	%	%	%	%	**%**	**%**
*Acidobacteria*	9.7	2	98	9.8	3	97	9.8	1	99	8.3	2	98	8.8	0	100
*Actinobacteria*	26.1	5	95	25.8	4	96	26.1	2	98	23.1	7	93	17.6	9	91
*Alphaproteobacteria*	10.9	4	96	7.4	4	96	6.5	2	98	14.3	3	97	27.7	8	92
*Bacteroidetes*	6.8	13	87	6.9	3	97	6.0	0	100	6.6	4	96	7.0	14	86
*Betaproteobacteria*	6.2	3	97	5.4	2	98	6.3	1	99	7.4	3	97	4.7	0	100
*Chloroflexi*	14.2	2	98	13.7	2	98	14.5	1	99	10.8	3	97	0.0	0	0
*Deltaproteobacteria*	4.1	7	93	3.8	1	99	3.6	1	99	4.3	2	98	5.1	0	100
*Gammaproteobacteria*	3.8	12	88	4.4	4	96	3.6	0	100	5.2	31	69	15.3	9	91
*Gemmatimonadetes*	2.9	4	96	3.5	1	99	5.1	1	99	1.7	2	98	0.0	0	0
*Planctomycetes*	3.7	2	98	3.8	2	98	3.3	1	99	2.8	4	96	5.8	0	100
*Tenericutes*	1.4	94	6	0.7	100	0	0.2	79	21	0.0	0	0	0.0	0	0
*TM7*	1.0	2	98	2.0	3	97	1.2	1	99	2.3	3	97	0.0	0	0
*Verrucomicrobia*	1.8	3	97	4.8	3	97	4.2	3	97	3.1	3	97	8.0	11	89
*Other*	7.3	4	96	7.9	8	92	9.5	1	99	10.2	4	96	0.0	0	0
Number of total degree of connection	59,706			80,836			29,606			70,916			704		

Quantitative report of taxa connection as a percentage (%) of the total degree (Tot), specifying the percentage of co-occurrence (+) and mutual exclusion (−) interaction observed for each taxon

**Table 2:** Evaluation of *Candidatus Phytoplasma* sp. OTU network interactions in grape root systems. Qualitative and quantitative comparison of *Candidatus Phytoplasma* sp. OTU interactions in grape root bacterial community networks

*Tenericutes* network interaction		157.11	161.49	420A	SO4	U.G.B.
Relative abundance (%)		5.6	0.3	0.00	6.1	0.0
Number of nodes		6	6	0	10	0
Node degree	Positive	54	0	0	24	0
	Negative	808	585	0	91	0
Edge target	Co-occurrence	23	0	0	0	0
	Mutual exclusion	329	245	0	30	0
Edge source	Co-occurrence	22	0	0	0	0
	Mutual exclusion	340	243	0	39	0

### Predicting bacterial functional profiles in the root tissues and rhizospheres of grafted and ungrafted Barbera plants

According to the analysis of the bacterial 16S rRNA gene dataset with the Tax4fun software package, a total of 61 ± 14% of OTUs can be mapped to KEGG organisms (Additional file 1: Table S16). The rootstock genotype did not affect the predicted metabolism of the root- and rhizosphere-associated bacterial communities (root: F_4,10_ = 2.6439, *p* = 0.065; rhizosphere: F_4,10_ = 1.3637, *p* = 0.251; Additional file 1: Figure S2 C and D), but it affected the beta-diversity of both the root and rhizosphere fractions (root: F_4,10_ = 6.9945, *p* = 0.001; rhizosphere: F_4,10_ = 4.4515, *p* = 0.001; Additional file 1: Figure S2 A and B). Also considering only the PGP key enzyme-encoding genes for biofertilization (nitrogen metabolism, phosphate solubilization and siderophore synthesis [21]) and biostimulation (indole acetic acid (IAA) production, 1-aminocyclopropane-1-carboxylate (ACC) deaminase activity and general PGP traits like VOCs production [22]), no rootstock effect was identified (root: F_4,10_ = 1.9637, *p* = 0.1004; rhizosphere: F_4,10_ = 1.2341, *p* = 0.3248; Additional file 1: Table S17 and Additional file 1: Figure S2 E and F). However, an analysis of the individual PGP traits showed that the enzyme-encoding genes for siderophore production, nitrogen metabolism and auxin (IAA) production were mostly spread throughout the bacterial communities associated with the root system, both in the root tissues and rhizosphere (Additional file 1: Figure S8). Except for IAA production, which had a 1.2-fold higher abundance in the estimated functional profile of the rhizosphere communities, the enzyme-encoding PGP genes were equally distributed between the bacterial communities associated with the root endosphere and the rhizosphere (Table 3). Looking inside each fraction, a significant rootstock effect was observed for only two of the PGP traits from the endophytic communities: ACC deaminase activity and phosphate solubilization pathways (Table 3 and Additional file 1: Figure S8B). The overall data show that the different rootstocks selected distinct bacterial populations, but not specific PGP functional traits within their bacterial communities.

**Table 3:** Functional predictions of grafted and ungrafted grape bacterial community plant growth-promoting (PGP) traits. Statistical results (*p* value) have been reported for each of the six PGP functional trait carried by grape bacterial communities to evaluate the effect of ‘Fraction’ (comparing root vs rhizosphere, *t* test) and the effect of ‘Rootstock’ (comparing rootstocks in both root and rhizosphere fractions, ANOVA). Significant values are reported in italics

Functional prediction of PGP traits	Fraction effect^a^	Rootstock effect^b^	
	Root vs rhizosphere	Root (df = 4,10)	Rhizosphere (df = 4,10)
ACC deaminase activity	0.291; t = 1.0937	*0.0006*; *F* **=** *12.77*	0.0538; F = 3.380
Auxin production	*0.038*; *t* = *2.141*	0.0720; F = 3.005	0.3988; F = 1.123
Nitrogen Metabolism	0.259; t = 1.0923	0.2955; F = 1.24	0.4616; F = 0.978
Phosphate solubilization	0.235; t = 1.2452	*0.0119*; *F = 5.681*	0.3389; F = 1.286
General PGP (VOCs production)	0.350; t = 0.9454	0.6981; F = 0.558	0.5278; F = 0.844
Siderophore synthesis	0.296; t = 1.0649	0.4891; F = 0.920	0.6096; F = 0.699

^a^*T* test statistical analysis was performed to compare the functional prediction of PGP traits in the two fractions. The *p* and *t* values have been reported to evaluate statistical significance

^b^One-way analysis of variance (ANOVA) was performed to evaluate the rootstock effect on the functional prediction of PGP traits in root and rhizosphere fractions. The *p* and F values have been reported to evaluate statistical significance

### Plant growth-promoting (PGP) potential of cultivable bacteria associated with grafted and ungrafted root systems

Cultivable bacteria associated with the root systems of the four different rootstocks and U.G.B. ranged from 10^6^ to 10^7^ CFUs/g in the root tissues and from 10^9^ to 10^10^ CFUs/g in the rhizosphere soil (Additional file 1: Table S18). Following a dereplication according to their ITS-PCR genotype, 68 of the initial 636 bacterial isolates were selected for PGP trait screening. The 68 isolates shared more than 98% 16S rRNA gene identity with their closest described relatives in the databases (Additional file 1: Table S19) and were ascribed to 17 genera in ten different families and five classes (Additional file 12: Table S20). *Enterobacteriaceae* dominated the whole collection (68% of the total isolates, with 52 and 83% of the isolates in the root endosphere and the rhizosphere, respectively), followed by *Pseudomonadaceae* (20% of the total isolates, with 26% and 14% in the root endosphere and the rhizosphere, respectively) and *Bacillaceae* (7% of the total isolates, but retrieved only from the root tissues). Interestingly, these families were represented by specific genera in the two root compartments. For instance, even though the most represented genus*, Enterobacter*, was found both in the rhizosphere and the root endosphere (56 and 21% of the total *Enterobacteriaceae*, respectively), specific genera for each of the two fractions were isolated from the collection. *Klebsiella* (16% of the total *Enterobacteriaceae*) was isolated from the rhizosphere only, while *Pantoea* (7%), *Rhanella* (11%) and *Serratia* (7%) were isolated from the root endosphere. *Pseudomonadaceae*, the second most abundant family, accounted only for 5% the isolates in the rhizosphere, but represented 29% of the isolates in the root endosphere. The distribution and abundance of the isolate type varied according to the rootstock type, with the rhizosphere having a more taxonomically homogenous cultivable community than the root endosphere (Additional file 1: Table S20).

Apart from ACC deaminase, which was used as a selective trait in the isolation process, the most common trait in the collection was IAA production, found in 85% of the isolates. The ‘biofertilization traits’, phosphate solubilization and siderophore production, were expressed in 62 and 53% of the isolates, respectively (Additional file 1: Table S21). While the distributions of the ‘biostimulation traits’ among rhizosphere and the root isolates were similar, the biofertilization traits were more represented among the rhizosphere isolates.

To assess the differences detected by cultivation dependent and independent approaches, the 16S rRNA gene sequences of the 68 bacterial isolates were compared with the 42 OTUs shared by the grafted and ungrafted grapevine root systems. Interestingly, 47 strains sequences (representing the 77% of the isolate collection) showed more than 97% identity with the two most abundant OTUs related to *Pseudomonas* (13%) and *Enterobacteriaceae* (10.2%). While the *Pseudomonas* OTU 2 matched (97% similarity) with isolates affiliated with the *Pseudomonas* genus in the cultivable fraction, several genera such as *Klebsiella*, *Citrobacter* and *Enterobacter* were affiliated (97% similarity) to OTU 4 of *Enterobacteriaceae*. The isolates corresponding to the two main OTUs presented potential PGP traits (Fig. 4e). All of the strains showed the potential to affect plant hormone balances, since they were capable of synthetizing ACC deaminase and producing IAA (100 and 91% of the isolates, respectively). Moreover, several isolates were capable of producing siderophores (64%) and enzymes involved in the solubilization of inorganic phosphate (72%), supporting their potential capacity to enhance iron and phosphate availability in the soil.

## Discussion

The root systems of the Barbera plants examined in this study, whether ungrafted or grafted on different rootstocks, recruited complex bacterial microbiomes that were largely composed of *Proteobacteria*, *Actinobacteria*, *Bacteroidetes*, *Cloroflexi* and *Acidobacteria*. Generally, diversification of the root microbiota begins in the rhizosphere fraction, where the root exudates recruit bacteria inhabiting the surrounding soil [6, 22–25]. Then, factors driven by the host-plant genotype generate a more distinct endophytic microbial community composed only of those microorganisms able to pass through the endodermis and pericycle to stably colonize the root tissues [23, 24, 26, 27].

We observed that the bacterial communities associated with each root type substantially differed in richness and diversity. Such a variability in the structure of the bacterial communities, especially in the endosphere, can be ascribed to (i) non-uniform colonization of the root system, (ii) slight variations in the plant physiology and growth stage, (iii) root exudation and even (iv) random events [26, 28]. Soil physicochemical properties, local bio-geographical factors and vineyard management procedures have been shown to affect the composition of root-associated communities throughout the grapevine lifespan [8, 25, 29–31]. The plant genotype is a known driver of the plant-associated microbiota structures, including those in the aerial endosphere and carposphere [32]. We demonstrated that the type of rootstock, independently of the scion cultivar and the soil type, is also a factor determining the specificity of the bacterial community in the grape root system compartments.

As grapevines are generally cultivated as scions (Barbera cultivar in our study) to be grafted on a rootstock, a large variety of scion-rootstock combinations are possible; this affects the productivity and quality of the grapes [18]. Plant genotype modulates the root metabolism, immune response and exudate composition, all of which in turn influence the activity and structure of the root microbiome [33]. Even small differences in the host genotype may alter the underground microbiome structure, impacting plant health [34]. Notably, the *V. vinifera* root systems in our study were endowed with complex microbiome structures tuned by the types of rootstock (different hybrids of *V. ripariae* × *V. berlandieri*) and plant species (*V. vinifera* vs. *V. ripariae* × *V. berlandieri*). Since grapevine rootstocks and their hybrids have been selected by breeders over the last century for their ability to provide ecological services to grape scions, such as tolerance to environmental factors (e.g. drought, soil limestone content and salinity) [13, 15, 35], the differences among the bacterial microbiome structures of grafted plants may be related to the rootstock species domestication, as has been observed in crop plant species such as wild plants and cultivars of rice [36]. In our study, the root system type determines the differential distribution of a subset of common community members between the rhizosphere and the root tissues and, at the same time, selects specific bacterial OTUs as biomarkers for the different root genotypes. The bacterial microbiomes of the different rootstocks were largely composed of *Alpha*- and *Gamma*-*Proteobacteria* and *Actinobacteria* that accounted for 42% of the relative abundance. The major phyla detected in our study do not differ considerably from those found in previous studies examining the bacterial microbiomes of grapevines (previous studies examined the root microbiome of different rootstocks, but not of ungrafted *V. vinifera* plants) in other geographical locations, suggesting that part of the bacterial microbiome associated with different rootstocks is conserved [8, 25, 29, 37, 38]. The ungrafted *V. vinifera* plants of the Barbera cultivar presented a root bacterial microbiome that was different from the rootstocks, especially in the root endosphere, supporting that diversity was driven by the plant species. An examination of other ungrafted *V. vinifera* cultivars would help to further disentangle such differences among species in the genus *Vitis* and among cultivars within *V. vinifera*.

The plant host and its associated microorganisms interact dynamically, defining a stable holobiont in which the partners cooperate to increase the fitness of the whole [1]. The functional capacity of microbiome is not equal to the sum of its individual components, since microbial species interact with each other and form a complex networks that significantly influence ecological processes and host adaptations [39]. The root genotype exerted a remarkable effect on the size and complexity of the grape root system networks, with the most extensive differences being between the grafted and ungrafted grape roots. We interpret the increased network size and complexity of the grafted grape root as an enhanced community organization with many bacterial interactions among the rhizosphere-root continuity. The higher number of positive interactions in grafted grape roots compared to ungrafted suggests that the enhanced cooperation is possibly driven by the increased metabolic exchanges. In contrast, the bacterial community networks of ungrafted roots were relatively simple, indicating a baseline level of interaction. The lack of complex root system networks in ungrafted plants might reflect an inactive or dormant state of many root-associated bacteria [40], without implicating a reduction of bacterial diversity [41]. Indeed, although ungrafted plants exhibited the lowest network complexity among the examined root systems, they showed high levels of diversity statistically comparable to those of the grafted plants.

The presence of interacting clusters of multiple species indicates the highly modularized structure of the grafted root systems, which result in increased interaction-network stability and, possibly, help the microbial community to resist biotic and abiotic stresses [42]. For instance, the *Tenericutes* nodes represented by relatively abundant reads of a *C. Phytoplasma* sp. closely related to the causal agent of bois noir disease in *V. vinifera*, *C. Phytoplasma solani* [20], were found in the root tissues of asymptomatic grafted plants. The transmission of phytoplasma by insect vectors normally occurs in the stems and leaves. However, phytoplasmas are capable of colonizing all of the plant organs, including the roots, as shown in garland chrysanthemums and *Vicia faba* [43, 44]. In the phytoplasma-inhabited rootstock, the surrounding bacterial communities developed negative interactions through a high number of mutual exclusions (Table 2), suggesting an active biocontrol that may prevent the disease from spreading, analogously to what may occur in the human gut [45]. It has been postulated that when threatened by an invading pathogen, the multi-trophic interactions among the plant, microbes, and environment are disrupted, and the native microbial community must be reconstructed [46]. During infection, phytoplasmas impact the composition of the endophytic microbial communities of diseased grapevines [47, 48], decreasing the overall bacterial diversity and increasing the abundance of bacteria affiliated with *Sphingobacterium sp.* [47]. Interestingly, we did not detect phytoplasma sequences in U.G.B., even though the origin was sympatric with the other plants included in the study, suggesting that the rootstocks may influence the ecology of the phytoplasma by becoming potential reservoirs of these cell-wall-less grape pathogens. An analysis of the microbial network interactions may help to clarify the host-pathogen interactions and provide new insights into the pathogen colonization process [49, 50].

Bacterial taxa that develop many interactions play relevant key roles within a microbiome [51]. Such taxa have large direct or indirect regulatory effects on other members of their community. All of the highly connected taxa in the grapevine root systems except *Tenericutes* were found in nodes that established positive interactions with the other components of the bacterial community. The *Actinobacteria* and *Alphaproteobacteria* showed the highest levels of connectivity, both in grafted and ungrafted roots, suggesting that they act as positive-interaction promoting hubs. Other highly connected taxa (i.e. *Chloroflexi*, *Gammaproteobacteria* and *Verrucomicrobia*) had different levels of connectivity in the grafted and ungrafted grape roots, indicating that they serve different functions in the bacterial communities of the different root systems. These bacteria have been observed in the root systems of other crops and were often associated with PGP activities such as protection and promotion of host growth under abiotic and biotic stresses [52, 53].

Considering the ability of bacteria to influence many aspects of plant health, the factors that shape the assembly and persistence of microbiota are of great interest to crop breeders and microbiologists [54]. Even though a significant proportion of bacterial diversity is driven by the root genotype, we observed that the predicted metabolisms are similar among the different root systems. This indicates that the potential functionalities and services of the bacterial microbiomes recruited by the different root systems are redundant and evenly spread among the root genotypes. We previously found that the PGP potential of cultivable bacteria in grape root systems was equally represented in root-associated bacterial communities, independent of the environmental factors such as the grape cultivar, country of the vineyard, climate, latitude, soil type and vineyard management [29]. Here, we further specify that functional redundancy is preserved in the variable bacterial microbiomes recruited by different root genotypes in the same soil, indicating that it is an intrinsic property of the studied vineyard soil. Indeed, functional redundancy, achieved through high diversity and vicariance, is crucial for maintaining a functioning ecosystem in agricultural systems [55].

For a selected core of the predicted PGP functional genes, the selection pressure of the different root genotypes was not mirrored by a different predicted functionality in the examined root compartments, suggesting that the potential ecological services were conserved both in the rhizosphere and the root endosphere. The conservation of PGP functional redundancy was confirmed in the cultivable bacterial fractions of the different grape root systems. Remarkably, the cultivable fraction we obtained mainly consisted of the *Enterobacteriaceae* and *Pseudomonadaceae* families, which were widely represented in the whole bacterial community of grape root as measured by the Illumina deep sequencing. These results are consistent with surveys on other grape root systems [8, 29, 56] that described these bacterial taxa as prominent members of the grape root holobiome. Despite the rhizosphere having higher bacterial density and diversity than the endosphere [57], we observed similar frequencies of PGP traits in both fractions of all of the root genotypes. All of the isolated bacteria presented at least two PGP traits potentially involved in the biofertilization or biostimulation of grapevine growth. Among the PGP features investigated, the abilities to produce auxin and ACC deaminase play an important role in microbe-interaction and plant adaptation. By inducing modification of the root system architecture [58], these morphological changes enhance the nutrient and water uptake of plants, resulting in a higher resistance to stresses [59, 60].

## Conclusion

The concept of *terroir* is determined by both the land characteristics of the vineyard and the plant-bacteria holobiome. The genotype of the grape root system strongly influences the selection and recruitment of bacterial components that may successively colonize the aboveground plant organs (i.e. stem, leaves, flower and fruit), influencing the quality of the fruit. As the root system is a key player in determining the plant-associated bacterial community, it contributes to the microbial *terroir* of the grapevine fruit and products. We proved that the bacterial communities in the root system of grape plants cultivated in the same soil and vineyard are significantly associated with the host genotype (rootstock type) and have different compositions and interactions. Despite selecting different bacterial components, grape root genotypes vicariate similar PGP traits carried by different bacteria that provide fundamental ecological services. Understanding the relationship between the bacterial community in the root system and its selection by the rootstock will provide helpful information for vineyard management and productivity, as well as elements to be considered in the comprehension of the microbial *terroir* of grapes.

## Methods

### Root system sampling and processing

The root systems of ungrafted Barbera grapevine plants (*V. vinifera* L., cv. Barbera) and Barbera grapevine rootstocks named 402A, 157.11, SO4 and 161.49 (*V. riparia* × *V. berlandieri*) were sampled in a vineyard at ‘Le Fracce’ farm (Oltrepo’ Pavese, Italy; latitude 45° 00′ 38.88′′ N and longitude 9° 08′ 25.44″ E), following the sampling design reported in Additional file 1: Figure S1. Features of the selected rootstock are reported in Additional file 1: Table S1 and Additional file Rootstock characterization. All of the selected grapes were cultivated in the same vineyard field, which was characterized by a clay-rich soil, and subjected to the same soil management practices for fertilization, irrigation and disease control [59]. The sampling was authorized by the owner, who is fully acknowledged in this paper, and no specific permissions were required for this activity. Grapevine roots were collected at 30–50 cm depth, where the root system was denser. Soil and root samples were collected under sterile conditions using sterile tools. The recovered samples were brought to the laboratory for further processing within 24 h from the time of sampling. The sampled roots with rhizosphere soil particles attached were placed in sterile tubes containing 9 mL of physiological solution (9 g/L NaCl). The tubes were vortexed for 5 min to detach the soil particles and then centrifuged at 4000 rpm for 5 min. The supernatant was discarded and the remaining soil fraction was used to represent the rhizosphere fraction. The clean roots were moved to a new tube and surface sterilized, as described in Cherif et al. [61]. Several washes with sterile water were performed to remove any trace of contaminants. The wash solution from the last rinse was cultured on PAF medium plates (10 g/L proteose peptone, 10 g/L hydrolyzed casein, 3 g/L MgSO_4_, 1.5 g/L K_2_HPO_4_, 10 mL/L glycerol and 15 g/L agar for solid medium) to determine the efficiency of sterilization.

### Metagenomic DNA extraction and metaphylogenomic analysis of 16S rRNA gene

The rhizosphere DNA was extracted from a 0.5-g sample using the PowerSoil® DNA Isolation Kit (MoBio Inc., CA, USA). One gram of sterilized root was crushed using liquid nitrogen to extract the DNA with a DNeasy Plant Max Kit (Qiagen). Illumina tag screening of the V3-V4 hypervariable regions of the 16S rRNA gene was performed on the DNA by Macrogen, Inc. (South Korea), using primers 341f and 785r [62]. The obtained sequences were analysed using a combination of the UPARSE v8 [63] and the QIIME v1.8 [64] software. Raw forward and reverse reads for each sample were assembled into paired-end reads considering a minimum overlapping of 50 nucleotides and a maximum of one mismatch within the region using the fastq-join algorithm (https://expressionanalysis.github.io/ea-utils/). The paired reads were then quality filtered discarding reads with a Phred quality score ≥ Q20 equivalent to a 0.01% error rate, the primer sequences have been removed and the individual sample files were merged in a single fasta file. This file was imported in UPARSE where operational taxonomic units (OTUs) of 97% sequence similarity were formed and chimeras were removed using both de-novo and reference-based detection. For reference chimera detection, the ‘Gold’ database containing the chimera-checked reference database in the Broad Microbiome Utilities (http://microbiomeutil.sourceforge.net/) was used. Taxonomy was assigned to the representative sequences of the OTUs in QIIME using UClust [63] and searching against the latest version of the Greengenes database [65]. Finally, an OTU table (i.e. a sample x OTU count matrix with a column containing the taxonomic affiliation of each OTU) was created. The OTU table and the phylogenetic tree were calculated with FastTree2 [66] using default parameters and the PyNast-aligned [64] representative sequences as an input. The OTU table and the phylogenetic tree were used as inputs for the subsequent analyses of alpha- and beta-diversity. The OTU table was log transformed for further statistical analysis [67]. A total of 579,974 high-quality merged paired-end reads with an average length of 445 bp were obtained from the 33 samples. Prior to further analysis, only the OTUs present in at least two-thirds of the replicates of each sample were selected. All of the samples analysed presented Good’s coverage values ranging from 0.96 to 1, capturing sufficient diversity with an adequate sequencing depth (Additional file 1: Figure S9).

### Bacterial diversity, taxonomic distribution and statistical analyses

OTUs present in less than two thirds of the replicates were discarded. A bipartite network analysis [68] of the bacterial community associated with the grape root system (root tissues and rhizosphere) and the bulk soil was performed using the QIIME script *make_bipartite_network.py* and was visualized in Cytoscape [69]. Nodes in the network corresponded to hosts and bacterial OTUs, with links indicating the presence of an OTU in the host. Bray-Curtis dissimilarity distance matrices were used to perform a principal coordinates analysis (PCoA), a canonical analysis of principal coordinates (CAP), and permutational analyses of variance (PERMANOVA) as described in Ramette [70] and Buttigieg and Ramette [71]. Statistical analyses were conducted in PRIMER v. 6.1, PERMANOVA+ for PRIMER routines [72] to test differences in the interaction, abundance and composition of the bacterial communities among the fixed and orthogonal explanatory variables, ‘Fraction’ (3 levels: root/rhizosphere/bulk) and ‘Rootstock Type’ (5 levels: U.G.B., 157.11, 161.49, SO4 and 420A). Prior to running PERMANOVA, we tested the homogeneity of the dispersions among the categorical variables using PERMDISP (F_11,33_ = 4.63; *p* = 0.23). Variance explained by the explanatory variable and their interaction has been calculated according with Nuccio et al. [73].

Non parametric statistical tests have been run to evaluate the taxonomical difference observed between root and rhizosphere (Wilcoxon rank test) and among rootstocks in the two fractions (Kruskal Wallis test) at phylum and genus level using the QIIME function *group_significance.py* [64]. For non-parametric multiple comparison test, a false discovery rate (FDR) has been applied [71]. Alpha diversity indices were calculated using the PAST software [74].

A linear discriminant analysis effect size (LEfSe) was applied to the OTU table (Wilcoxon *p*-value: 0.05, LDA > 2; http://huttenhower.sph.harvard.edu/galaxy/) to identify the discriminant bacterial clade of rootstock in both the root and rhizosphere fractions, according to the method described elsewhere [75]. A Similarity Percentages (SIMPER) analysis was performed with PRIMER 6 to explore the dissimilarities between the Fraction and Rootstock Type factors. Summarized taxa tables at the phylum and genera levels were used to investigate the phylogenetic groups that contribute to the dissimilarity. Unclassified OTUs amounting to less than 3% of the relative abundance in the root system were discarded from the analysis, following a previous study [76].

The OTUs shared among different rootstocks and fractions were defined by a Venn-diagram analysis using the software available at http://bioinformatics.psb.ugent.be.

The OTU table was also used to predict the functional potential of bacterial communities using Tax4Fun [77]. Since Tax4Fun only recognized taxonomical data from the SILVA database, the OTUs previously obtained (as described in the paragraph ‘Metagenomic DNA extraction and metaphylogenomic analysis of 16S rRNA gene*’*) were taxonomically reassigned using the command *assign_taxonomy.py* in the QIIME pipeline and the SILVA 119 database [78]. Once obtained SILVA119-based OTUs table, Tax4Fun generated a relative abundance of KEGG orthology (KO) groups associated with each sample depending on matches between the representative sequences from each SILVA119-based OTUs and the KEGG organisms [79, 80]. The taxonomic profile of KEGG organisms obtained by the SILVA-based OTUs was normalized by the 16S rRNA copy number (obtained from NCBI genome annotations).

The fraction of OTUs that could not be mapped to KEGG organisms is reported in supplementary material and ranging from 18 to 67% (Additional file 1: Table S15).

KEGG orthologs (KO) involved in the PGP mechanisms (auxin production, nitrogen metabolism, phosphate solubilization, siderophore synthesis, ACC deaminase activity and general PGP activity) were extracted using the KEGG database and several reviews [81–83].

### Co-occurrence network analysis

A co-occurrence network analysis was performed for each microbiome associated with the rhizosphere and endophyte of each rootstock to explore the significant relations among the OTUs [84], using the routine CoNet in Cytoscape 3.4 [85]. To build the network, we filtered out the OTUs with frequencies less than 0.05, then combined an ensemble of the Pearson and Spearman correlation coefficients, and the Bray-Curtis (BC) and Kullback-Leibler (KLD) dissimilarity indices [85, 86]. To compute the statistical significance of the co-occurrence and mutual exclusions, we computed the edge-specific permutation and bootstrap score distributions with 1000 iterations. We re-normalized the data in each permutation, providing a null distribution that captured the similarity introduced by the composition. Then, we computed the *p* value, as above, by z-scoring the permuted null and bootstrap confidence interval using the pooled variance [87]. The Clustering coefficients, neighbourhood connectivity distribution, betweenness centrality, topological coefficients and modularity index were calculated as the most important statistical descriptors of the network [88]. To visualize the network, we used Gephi [89]. Statistical differences between the degrees of connection in the co-occurrence networks for ungrafted and grafted grape root system nodes were analysed using the R package MASS; a generalized linear model using the Poisson error distribution was used for the count data [90]. To visualize the taxonomy of the most connected nodes (Fig. 5), we selected the nodes with a connection frequency of more than 75%.

### Isolation, cultivation and molecular identification of bacteria isolates

Either 1 g of the rhizosphere soil or 1 g of the sterilized root obtained as described above was used as inoculum for the ACC-deaminase enrichment culture, as described by Penrose and Glick [91]. A total of 636 colonies were randomly picked from the isolation plates, and the bacteria were propagated three times on a PAF medium. The purified strains were frozen in 25% glycerol at − 80 °C for later use. The redundancy of the microbial collection was reduced by a dereplication procedure, based on the heterogeneity of the 16S-23S rRNA Internal Transcribed Spacers (ITS) measured by ITS-PCR fingerprinting [92]. A total of 68 ITS haplotypes were identified by 16S rRNA sequencing. The gene was amplified using universal 27F and 1492R primers in a PCR reaction under previously reported conditions [93]. The sequencing was performed by Macrogen Inc. (South Korea). The sequences were edited in Chromas lite 2.01 (http://technelysium.com.au/wp/) and subjected to BLAST (http://blast.ncbi.nlm.nih.gov/Blast.cgi). The OTU/isolate sequences were aligned using the SILVA Incremental Aligner (SINA, [94]), where the conserved blocks were selected with the Gbloks software [95]. The phylogenetic analysis was performed using Molecular Evolutionary Genetic Analysis MEGA7 [96] to apply the neighbor-joining method [97] to 1000 replicates as a bootstrap test.

To evaluate and quantify the presence of bacterial isolates in the total community, 16S rRNA sequences of the isolates were blasted against the most abundant OTU sequences (OTU2, OTU4, OTU7 and OTU11). When the percent identity was above 97% with coverage over 95% of the OTU sequence, the isolate sequences were aligned and a phylogenetic analysis was performed using the method described above.

### In vitro characterization of the PGP potential of ACC deaminase cultivable bacteria

The 68 selected isolates (showing different ITS haplotypes) were screened in vitro to evaluate their plant growth-promoting (PGP) activity score. The protocol described by Bric et al. [98] was followed to determine the indole acetic acid (IAA) production. The presence of IAA in the culture supernatant was determined spectrophotometrically at 530 nm. Pure IAA (Sigma-Aldrich Co., Italy) was used as the standard and uninoculated media served as the control. The mineral P-solubilizing ability of the strains was determined on Pikovskaya’s liquid medium amended with 0.5% tricalcium phosphate [Ca_3_(PO_4_)_2_] as inorganic P [99]. Siderophore release was detected by the formation of orange halos on Chrome Azural S (CAS) agar plates after incubating for 7 days at 30 °C, as described elsewhere [100].

### Nucleotide sequence accession numbers

The sequences of the partial 16S rRNA genes for isolates were deposited in the GeneBank database under the accession numbers KY810694–KY810761 and HF562860–HF562905. The sequence reads were deposited in the NCBI SRA database under the BioProject ID: PRJNA378357 (accession numbers SRR5318246–SRR5318255).

## Additional file

Additional file 1: Table S1. Information about rootstocks and ungrafted grape, **Table S2**. Number of sequences, total OTUs and diversity indices, **Table S3**. Pair-wise post hoc test comparison of rootstock bacterial communities associated to grape root system, **Table S4**. Main test comparison of bacterial’ beta-diversity **Table S5**. (A) Cross Validation of ‘Fraction’ × ‘Rootstock’ groups. (B) Estimation of beta-diversity variation’ components, **Table S6**. Rootstock’ specific and shared OTUs, **Table S7**. Phylogenetic affiliation of OTUs, **Table S8**. Linear discriminant analysis Effect Size of bacterial OTUs in root and rhizosphere, **Table S9**. Main phyla and genera contributing to the dissimilarity among rootstocks, **Table S10**. Phyla and genera contribution to rootstocks dissimilarity, **Table S11**. Relative abundance and phylogenetic affiliation of OTUs’ core, **Table S12**. Number of sequence and relative abundance of rootstock specific OTUs, **Table S13**. Topological property of co-occurring bacterial networks, **Table S14**. Nodes tables of co-occurring bacterial networks, **Table S15**. Edge tables of co-occurring bacterial network, **Table S16**. Quality statistical analysis of Tax4Fun results, **Table S17**. KEGG enzyme-encoding gene for PGP traits and their abundances in the predicting functional profiles of rootstock bacterial communities, **Table S18**. Number of cultivable bacteria expressed as colony-forming unit per gram of sample, **Table S19**. Phylogenetic identification of one representative bacterial isolates for each ITS-haplotype, **Table S20**. Phylogenetic identification of bacteria isolates for each rootstock, **Table S21**. Screening of bacterial isolates for PGP activities, **Figure S1**. Sampling scheme, **Figure S2**. Beta-diversity and functional prediction,** Figure S3**. Alpha-diversity, **Figure S4**. Relative abundance of genera belonging to *Actinobacteria*, *Alphaproteobacteria* and *Gammaproteobacteria*, **Figure S5**. Nodes degree distribution, **Figure S6**. Degree distribution of node in the third percentile, **Figure S7**. Phylogenetic tree of ‘*Candidatus Phytoplasma*’, **Figure S8**. Relative abundances of predicted PGP traits, **Figure S9**. Rarefaction curve and Good’s coverage values. (DOCX 1603 kb)

## Abbreviations

157.11: Barbera grape (*V. vinifera* sp.) grafted on 157.11 rootstock (*V. riparia* × *V. berlandieri*)
161.49: Barbera grape (*V. vinifera* sp.) grafted on 161.49 rootstock (*V. riparia* × *V. berlandieri*)
420A: Barbera grape (*V. vinifera* sp.) grafted on 420A rootstock (*V. riparia* × *V. berlandieri*)
SO4: Barbera grape (*V. vinifera* sp.) grafted on SO4 rootstock (*V. riparia* × *V. berlandieri*)
ACC: 1-Aminocyclopropane-1-carboxylate
CAP: Canonical analysis of principal coordinates
CFU: Colony-forming unit
IAA: Indole acetic acid
ITS-PCR: 16S-23S rRNA gene internal transcribed spacer polymerase chain reaction
KEGG: Kyoto encyclopaedia of genes and genomes
LEfSe: Linear discriminant analysis effect size
OTU: Operational taxonomic unit
PCoA: Principal coordinate analysis
PCR: Polymerase chain reaction
PERMANOVA: Permutational multivariate analysis of variance
PERMDISP: Distance-based test for homogeneity of multivariate dispersions
PGP: Plant growth promoting
QIIME: Quantitative insights into microbial ecology
qPCR: Quantitative polymerase chain reaction
rRNA: Ribosomal RNA
SIMPER: Similarity percentages
U.G.B: Ungrafted Barbera grape (*V. vinifera* sp.)
