# Supplementary material for: Grapevine rootstocks shape underground bacterial microbiome and networking but not potential functionality
Source: Microbiome. 2018 Jan 3;6:3. doi: 10.1186/s40168-017-0391-2 (PMC5751889; doi:10.1186/s40168-017-0391-2)
Supplement: Additional file 1: Table S1. — Information about rootstocks and ungrafted grape, Table S2. Number of sequences, total OTUs and diversity indices, Table S3. Pair-wise post hoc test comparison of rootstock bacterial communities associated to grape root system, Table S4. Main test comparison of bacterial’ beta-diversity Table S5. (A) Cross Validation of ‘Fraction’ × ‘Rootstock’ groups. (B) Estimation of beta-diversity variation’ components, Table S6. Rootstock’ specific and shared OTUs, Table S7. Phylogenetic affiliation of OTUs, Table S8. Linear discriminant analysis Effect Size of bacterial OTUs in root and rhizosphere, Table S9. Main phyla and genera contributing to the dissimilarity among rootstocks, Table S10. Phyla and genera contribution to rootstocks dissimilarity, Table S11. Relative abundance and phylogenetic affiliation of OTUs’ core, Table S12. Number of sequence and relative abundance of rootstock specific OTUs, Table S13. Topological property of co-occurring bacterial networks, Table S14. Nodes tables of co-occurring bacterial networks, Table S15. Edge tables of co-occurring bacterial network, Table S16. Quality statistical analysis of Tax4Fun results, Table S17. KEGG enzyme-encoding gene for PGP traits and their abundances in the predicting functional profiles of rootstock bacterial communities, Table S18. Number of cultivable bacteria expressed as colony-forming unit per gram of sample, Table S19. Phylogenetic identification of one representative bacterial isolates for each ITS-haplotype, Table S20. Phylogenetic identification of bacteria isolates for each rootstock, Table S21. Screening of bacterial isolates for PGP activities, Figure S1. Sampling scheme, Figure S2. Beta-diversity and functional prediction, Figure S3. Alpha-diversity, Figure S4. Relative abundance of genera belonging to Actinobacteria, Alphaproteobacteria and Gammaproteobacteria, Figure S5. Nodes degree distribution, Figure S6. Degree distribution of node in the third percentile, Figure S7. Phylogenetic tree of ‘Can [file 40168_2017_391_MOESM1_ESM.docx]

**ADDITIONAL 1 FILES to manuscript**

**Rootstocks shape grapevine underground bacterial microbiome and networking but not potential functionality**

Ramona Marasco^1#^, Eleonora Rolli^2#^, Marco Fusi^1^, Grégoire Michoud^1^, Daniele Daffonchio^1,2*^

^1^ Biological and Environmental Sciences and Engineering Division, King Abdullah University of Science and Technology, Thuwal 23955-6900, Kingdom of Saudi Arabia.

^2^ Department of Food Environmental and Nutritional Sciences, Università degli Studi di Milano, 20133, Milano, Italy.

^#^ Equal contribution

^*^ Authors for correspondence: Daniele Daffonchio, daniele.daffonchio@kaust.edu.sa

**ADDITIONAL FILE ON ROOTSTOCK CHARACTERISTICS**

The studied rootstocks are among the most common ones used in recent years to be nested with the Barbera scion [1]. The selected rootstocks (157.11, 161.49, SO4 and 420A) have been obtained by the cross of two *Vitis* species, *V. riparia* and *V. berlandieri*. They were selected following experimental field trials of the Scion × Rootstock combination to test phenotypic response to mineral nutrition, soil adaptability, resistance to abiotic and biotic stresses, vegetative parameters and quality fruit and yield (Additional file Table S1). Their genetic diversity has been recently studied [2,3]. A genotyping study performed by AFLP (Amplified Fragment Length polymorphism) on 19 grape rootstocks, showed that the two 420A and SO4 rootstocks were close to Fercal rootstock, a complex cross clone ancestor of *V. berlandieri* [3]. SSR analysis (Simple Sequence Repeat) on 96 grape accessions, mainly rootstocks, was performed by Jahnke and colleagues 2014 [2]. The study revealed that, despite their genetic diversity, the rootstocks SO4, 420A and 161.49 shared some similarities. They are genetically classified close to other rootstocks of the cross *V. berlandieri* × *V. riparia*. Their genetic similarity is due to their common origin from the Teleki’s seedlings collection [2]. Moreover, the scion-rootstock interaction seems to be more complex than thought before.

**References**

1. Achilli E, Labe A, Venturi A. Il vigneto piacentino: aspetti agronomici. Vignetivini. 2001;28:42–5.

2. Jahnke G, Májer J, Szőke B, Varga P, Kocsisné Molnár G, Tarczal E, et al. Analysis of grape rootstocks by microsatellite markers. Acta Hortic. 2014;1046:617–26.

3. Sabir A, Dogan Y, Tangolar S, Kafkas S. Analysis of genetic relatedness among grapevine rootstocks by AFLP (Amplified Fragment Length Polymorphism) markers. J. Food, Agric. Environ. 2010;8:210–3.

**ADDITIONAL FILE TABLES**

**Additional file Table S1.** Information about rootstock selected in this study and ungrafted grape. This information is based on written reports throughout Europe and the New World available in the web.

| **Rootstock Type** | **Rootstock germplasm** | **Phylloxera resistance** | **Scion**  **Vigor** | **Tolerance** | | | **Root system** |
| --- | --- | --- | --- | --- | --- | --- | --- |
|  |  |  |  | **Drought** | **Wet soil** | **Salt** |  |
| 157.11 | *V. berlandieri* × *V. Riparia* | High | Medium | Medium | Medium | Medium | Semi-deep |
| 161.49 | *V. berlandieri* × *V. Riparia* | High | Medium | Medium | Low | Medium | Semi-deep |
| SO4 | *V. berlandieri* × *V. Riparia* | High | Medium/High | Low | Medium | Low | Semi-superficial |
| 420A | *V. berlandieri* × *V. Riparia* | High | Medium/Low | Medium | Low | Low | Semi-deep |
| U.G.B | *V. vinifera* | n.d. | n.d. | n.d. | n.d. | n.d. | n.d. |

**Additional file Table S2.** Number of sequences, total OTUs and diversity indices expressed as average and standard deviation in the selected rootstocks, both in the root and rhizosphere fractions. Letter in parenthesis indicated the one-way analysis of variance (ANOVA) performed for each index analyzed (Tukey's Multiple Comparison Test, *p*<0.05).

| **Index** |  | **Fraction** |  |
| --- | --- | --- | --- |
|  | **Root** | **Rhizosphere** | **Bulk** |
| OTUs | 163 ± 59 (a) | 918 ± 295 (b) | 1533 ± 50 (c) |
| Sequence | 10497 ± 7520 (a) | 21704 ± 6168 (b) | 23024 ± 2251 (b) |
| Dominance | 0.23 ± 0.221 (a) | 0.03 ± 0.026 (b) | 0.005 ± 0.001 (ab) |
| Simpson | 0.77 ± 0.221 (a) | 0.97 ± 0.026 (b) | 0.995 ± 0013 (ab) |
| Shannon | 2.799 ± 1.311 (a) | 4.998 ± 0.772 (b) | 6.216b ± 0.026 (b) |
| Evenness | 0.166 ± 0.133 (a) | 0.191 ± 0.081 (a) | 0.327 ± 0.004 (a) |

| **Index** | **Root** | | | | |
| --- | --- | --- | --- | --- | --- |
|  | **157.11** | **161.49** | **420A** | **SO4** | **U.G.B.** |
| OTUs | 180 ± 18 (a) | 183 ± 24 (a) | 60 ± 6 (b) | 183 ± 47 (a) | 212 ± 5 (a) |
| Sequence | 8468 ± 4524 (a) | 4612 ± 1018 (a) | 22708 ± 695 (b) | 6269 ± 4416 (a) | 10428 ± 6767 (a) |
| Dominance | 0.252 ± 0.209 (ab) | 0.053 ± 0.005 (a) | 0.5285 ± 0.104 (b) | 0.034 ± 0.014 (ac) | 0.281 ± 0.209 (ab) |
| Simpson | 0.748 ± 0.209 (ab) | 0.95 ± 0.005 (a) | 0.4715 ± 0.104 (b) | 0.966 ± 0.014 (ac) | 0.719 ± 0.209 (ab) |
| Shannon | 2.556 ± 0.63 (ab) | 3.84 ± 0.038 (a) | 0.881 ± 0.061 (b) | 4.147 ± 0.33 (a) | 2.572 ± 1.23 (ab) |
| Evenness | 0.078 ± 0.035 (a) | 0.258 ± 0.05 (bc) | 0.040 ± 0.003 (a) | 0.354 ± 0.023 (b) | 0.102 ± 0.117 (ac) |

| **Index** | **Rhizosphere** | | | | |
| --- | --- | --- | --- | --- | --- |
|  | **157.11** | **161.49** | **420A** | **SO4** | **U.G.B.** |
| OTUs | 732 ± 105 (ab) | 963 ± 63 (b) | 540 ± 105 (a) | 1348 ± 91 (c) | 1007 ± 122 (b) |
| Sequence | 24242 ± 5366 (a) | 22414 ± 4118 (a) | 19596 ± 13385 (a) | 22496 ± 1181 (a) | 19773 ± 4048 (a) |
| Dominance | 0.067 ± 0.039 (a) | 0.028 ± 0.005 (ab) | 0.029 ± 0.012 (ab) | 0.011 ± 0.009 (b) | 0.013 ± 0.002 (b) |
| Simpson | 0.933 ± 0.039 (a) | 0.972 ± 0.005 (ab) | 0.971 ± 0.012 (ab) | 0.989 ± 0.009 (b) | 0.987 ± 0.002 (b) |
| Shannon | 3.94 ± 0.74 (a) | 5.01 ± 0.05 (ab) | 4.69 ± 0.37 (ac) | 5.87 ± 0.386 (b) | 5.48 ± 0.17 (bc) |
| Evenness | 0.079 ± 0.038 (a) | 0.157 ± 0.004 (ab) | 0.206 ± 0.041 (b) | 0.272 ± 0.081 (b) | 0.241 ± 0.035 (b) |

**Additional file Table S3.** Pair-wise post hoc test comparison using PERMANOVA on the Bray Curtis similarity matrices in the (**A**) total bacterial community to evaluate the ‘Fraction’ effect and in the two root compartments (**B**, root and **C**, rhizosphere) to evaluate the ‘Rootstock’ effect. Asterisks indicated significant differences.

| **(A) Fraction effect** |  |  |
| --- | --- | --- |
| **Fraction** | **T** | ***p*** |
| Root, Rhizosphere | 4.4852 | 0.001* |
| Root, Bulk soil | 3.4097 | 0.001* |
| Rhizosphere, Bulk soil | 2.3589 | 0.002* |

| **(B) Rootstock effect in Root**  **F_4,10_=** **6.9945, *p*=0.001** | | |  | **(C) Rootstock effect in Rhizosphere**  **F_4,10_=** **4.4515, *p*=0.001** | | |
| --- | --- | --- | --- | --- | --- | --- |
| **Rootstock** | **t** | ***P*** |  | **Rootstock** | **t** | ***p*** |
| 157.11, 161.49 | 2.5586 | 0.0119* |  | 157.11, 161.49 | 1.9889 | 0.0304* |
| 157.11, 420A | 3.0217 | 0.0075* |  | 157.11, 420A | 1.7855 | 0.0496* |
| 157.11, U.G.B. | 2.6053 | 0.0110* |  | 157.11, U.G.B. | 2.7591 | 0.0097* |
| 157.11, SO4 | 2.1632 | 0.0228* |  | 157.11, SO4 | 2.081 | 0.0304* |
| 161.49, 420A | 3.5545 | 0.0036* |  | 161.49, 420A | 2.0118 | 0.0354* |
| 161.49, U.G.B. | 2.554 | 0.0124* |  | 161.49, U.G.B. | 2.5314 | 0.0125* |
| 161.49, SO4 | 2.2792 | 0.0193* |  | 161.49, SO4 | 1.8062 | 0.0459* |
| 420A, U.G.B. | 2.9618 | 0.0074* |  | 420A, U.G.B. | 2.5189 | 0.0134* |
| 420A, SO4 | 2.6048 | 0.0116* |  | 420A, SO4 | 1.763 | 0.0562 |
| U.G.B., SO4 | 2.0672 | 0.0270* |  | U.G.B., SO4 | 2.0353 | 0.0326* |

**Additional file Table S4.** Main test comparison of the bacterial communities’ beta-diversity using two-way PERMANOVA.

| **Factor** | **Df** | **MS** | **Pseudo-F** | ***p*** |
| --- | --- | --- | --- | --- |
| Fraction | 1 | 28433.0 | 47.33 | 0.001 |
| Rootstock | 4 | 3811.8 | 6.35 | 0.001 |
| Fraction X Rootstock | 4 | 3078.0 | 5.12 | 0.001 |

**Additional file Table S5.** (**A**) Cross Validation of the proposed group (‘Fraction’ × ‘Rootstock’) calculated using Canonical Analysis of Principal coordinates with the PRIMER software. (**B**) PERMANOVA estimated components of variation in the beta-diversity index calculated using the PRIMER software.

**(A)** Canonical Analysis of Principal coordinates cross-validation (choice of m: 7; No. of permutations used: 999; delta_1^2: 0.99501 P=0.001).

| **Group** | **157-E** | **161-E** | **420A-E** | **UGB-E** | **SO4-E** | **157-R** | **161-R** | **420A-R** | **UGB-R** | **SO4-R** | **Bulk** |
| --- | --- | --- | --- | --- | --- | --- | --- | --- | --- | --- | --- |
| **157-E** | **3** | 0 | 0 | 0 | 0 | 0 | 0 | 0 | 0 | 0 | 0 |
| **161-E** | 0 | **3** | 0 | 0 | 0 | 0 | 0 | 0 | 0 | 0 | 0 |
| **420A-E** | 0 | 0 | **3** | 0 | 0 | 0 | 0 | 0 | 0 | 0 | 0 |
| **UGB-E** | 0 | 0 | 0 | **3** | 0 | 0 | 0 | 0 | 0 | 0 | 0 |
| **SO4-E** | 0 | 0 | 0 | 0 | **3** | 0 | 0 | 0 | 0 | 0 | 0 |
| **157-R** | 0 | 0 | 0 | 0 | 0 | **3** | 0 | 0 | 0 | 0 | 0 |
| **161-R** | 0 | 0 | 0 | 0 | 0 | 0 | **3** | 0 | 0 | 0 | 0 |
| **420A-R** | 0 | 0 | 0 | 0 | 0 | 0 | 0 | **3** | 0 | 0 | 0 |
| **UGB-R** | 0 | 0 | 0 | 0 | 0 | 0 | 0 | 0 | **3** | 0 | 0 |
| **SO4-R** | 0 | 0 | 0 | 0 | 0 | 0 | 0 | 0 | 0 | **3** | 0 |
| **Bulk** | 0 | 0 | 0 | 0 | 0 | 0 | 0 | 0 | 0 | 0 | **3** |

**(B)** PERMANOVA

| **Factor** | **Estimate** | **Sq*.* root** | **%** |
| --- | --- | --- | --- |
| Fraction | 1623.40 | 40.292 | 46.4 |
| Rootstock | 349.18 | 18.686 | 10 |
| Fraction X Rootstock | 407.55 | 20.188 | 11.6 |
| Unexplained variance (residual) | 1120.60 | 33.475 | 32 |

**Additional file Table S6**. Shared OTUs of ungrafted and grafted Barbera grapes in **(A)** root and **(B)** rhizosphere compartments. **(C)** Overlaps between shared OTUs in root and rhizosphere of all the samples (ungrafted and grafted plants).

| **(A) Root** | **Number of OTUs** | **Specific OTUs** |
| --- | --- | --- |
| 157.11 | 214 | 50 |
| 161.49 | 216 | 44 |
| 420A | 229 | 1 |
| SO4 | 74 | 63 |
| UGB | 263 | 85 |
| Overall number of unique OTUs | 466 |  |
| Shared OTUs | 50 |  |
|  |  |  |
| **(B) Rhizosphere** | **Number of OTUs** | **Specific OTUs** |
| 157.11 | 895 | 65 |
| 161.49 | 1132 | 107 |
| 420A | 1546 | 39 |
| SO4 | 683 | 124 |
| UGB | 1227 | 346 |
| Overall number of unique OTUs | 2097 |  |
| Shared OTUs | 358 |  |

| **(C) Shared OTUs in the root system** | **Number of OTUs** | **Specific OTUs** |
| --- | --- | --- |
| Root | 50 | 8 |
| Rhizosphere | 358 | 316 |
| Overall number of unique OTUs | 366 |  |
| Shared OTUs | 42 |  |

**Additional file Table S7.** Phylogenetic affiliation at phylum, order, family and genus levels (attached Marasco et al 2017_Additional files Table S7)

**Additional file Table S8.** Linear discriminant analysis Effect Size (LEfSe) of bacterial OTUs in root (A) and rhizosphere (B) of grafted and ungrafted grape root system (attached Marasco et al 2017_Additional files Table S8).

**Additional file Table S9.** Similarity percentages (SIMPER) analysis determines the **(A)** phyla and **(B)** genera contributions to the dissimilarity among rootstocks in root and rhizosphere fractions. In the upper part of the table the rootstock pairwise comparison of average dissimilarity percentage has been reported. In the lower part, the overall top three phyla/genera contributing to the pairwise dissimilarity were listed, reporting in parenthesis their relative contribution to the observed dissimilarity expressed as percentage.

**(A)** SIMPER analysis determined the phyla contributions in root (upper table) and rhizosphere (lower table).

^#^ Rootstock-pairs showing dissimilarity in phyla distribution higher than 10%

^†^ Rootstock-pair showing the lowest dissimilarity observed in phyla distribution

**(B)** SIMPER analysis determined the genera contributions in root (upper table) and rhizosphere (lower table).

^#^ Rootstock-pairs showing dissimilarity in genera distribution higher than 25%

^†^ Rootstock-pair showing the lowest dissimilarity observed in genera distribution

**Additional file Table S10.** Similarity percentages (SIMPER) analysis determined the phyla (**A** and **B**) and genera (**C** and **D**) contributions to the dissimilarity among rootstocks in root and rhizosphere fractions (attached Marasco et al 2017_Additional files Table S10).

**Additional file Table S11**. Relative abundance and phylogenetic affiliation of OTUs’ core of ungrafted and grafted Barbera grapes in **(A)** root and **(B)** rhizosphere compartments, and in **(C)** the entire root system. See attached Excel file: Marasco et al 2017_Additional files Table S11.

**Additional file Table S12**. Number of sequence and relative abundance of ungrafted and grafted Barbera grapes specific OTUs in **(A)** root and **(B)** rhizosphere fractions. See attached Excel file: Marasco et al 2017_Additional files Table S12.

**Additional file Table S13.** Topological property of co-occurring bacterial networks of grafted and ungrafted grape root systems (root and rhizosphere) calculated using the statistical Cytoscape package.

| **Network parameters** | **Rootstock** | | | | |
| --- | --- | --- | --- | --- | --- |
|  | **157.11** | **161.49** | **420A** | **SO4** | **U.G.B.** |
| Modularity | 0.523 | 0.616 | 0.590 | 0.739 | 0.898 |
| Connected component | 45 | 66 | 43 | 62 | 16 |
| Clustering coefficient | 0.540 | 0.641 | 0.695 | 0.763 | 0.152 |
| Average neighbors | 57.576 | 64.617 | 39.549 | 64.410 | 3.502 |
| Average path length | 2.994 | 3.216 | 2.767 | 3.331 | 5.411 |
| Network centralization | 0.117 | 0.076 | 0.145 | 0.100 | 0.058 |
| Network heterogeneity | 1.119 | 0.893 | 0.894 | 0.930 | 0.791 |
| Number nodes | 1037 | 1251 | 750 | 1101 | 201 |
| Network diameter | 8 | 8 | 6 | 8 | 12 |
| Network density | 0.056 | 0.052 | 0.053 | 0.059 | 0.018 |
| Degree distance | 180 | 160 | 149 | 175 | 16 |
| Interaction | 29853 | 40418 | 14831 | 35458 | 352 |
| Co-occurrence | 28073 | 38805 | 14053 | 34962 | 327 |
| Mutual exclusion | 1078 | 1613 | 778 | 496 | 25 |

**Additional file Table S14.** Nodes tables of co-occurring bacterial network analysis for grafted and ungrafted grape root systems. See attached Excel file: Marasco et al 2017_Additional files Table S14.

**Additional file Table S15.** Edge tables of co-occurring bacterial network analysis for grafted and ungrafted grape root systems. See attached Excel file: Marasco et al 2017_Additional files Table S15.

**Additional file Table S16**. Quality statistical analysis of Tax4Fun results. The portion of OTUs that cannot be mapped to KEGG organisms (FTU) is reported for each sample. FTU portion is showed in root (E) and rhizosphere (R) fractions for ungrafted (U.G.B) and grafted (157, 161, 420A and SO4) grape.

| **Fraction** | **Rootstock** | **Samples** | **FTU** |
| --- | --- | --- | --- |
| Root | 157.11 | 157E-E1 | 0.483300301 |
|  |  | 157E-E2 | 0.477278015 |
|  |  | 157E-E3 | 0.663898535 |
|  | 161.46 | 161E-E1 | 0.67063332 |
|  |  | 161E-E2 | 0.406289671 |
|  |  | 161E-E3 | 0.639874353 |
|  | 420A | 420A-E1 | 0.559043148 |
|  |  | 420A-E2 | 0.17776927 |
|  |  | 420A-E3 | 0.501820143 |
|  | U.G.B. | U.G.B.-E1 | 0.41181734 |
|  |  | U.G.B.-E2 | 0.593004018 |
|  |  | U.G.B.-E3 | 0.592360621 |
|  | SO4 | SO4-E1 | 0.444951993 |
|  |  | SO4-E2 | 0.343797856 |
|  |  | SO4-E3 | 0.592004381 |
| Rhizosphere | 157.11 | 157-R1 | 0.270215213 |
|  |  | 157-R2 | 0.551775077 |
|  |  | 157-R3 | 0.669154229 |
|  | 161.46 | 161-R1 | 0.324063342 |
|  |  | 161-R2 | 0.653556675 |
|  |  | 161-R3 | 0.340207419 |
|  | 420A | 420A-R1 | 0.234344152 |
|  |  | 420A-R2 | 0.57507801 |
|  |  | 420A-R3 | 0.205813953 |
|  | U.G.B. | U.G.B.-R1 | 0.590235506 |
|  |  | U.G.B.-R2 | 0.534587554 |
|  |  | U.G.B.-R3 | 0.516915654 |
|  | SO4 | SO4-R1 | 0.548608468 |
|  |  | SO4-R2 | 0.531565998 |
|  |  | SO4-R3 | 0.677909031 |

**Additional file Table S17**. List of the selected KEGG enzyme-encoding gene for PGP traits involved in biofertilization (nitrogen metabolism, phosphate solubilization and siderophore synthesis) and biostimulation (auxin production activity, ACC deaminase activity and PGP activity such as VOCs production) and their abundances in the predicting functional profiles of bacterial communities associated to root and rhizosphere of grafted and ungrafted grape plants. See attached Excel file: See attached Excel file: Marasco et al 2017_Additional files Table S17.

**Additional file Table S18.** Number of cultivable bacteria expressed as average ± standard deviation of colony-forming unit (CFU) *per* gram sample (root tissues or rhizospheric soil).

| **Fraction** | **Rootstock** | **CFU/g** |  | **Fraction** | **Rootstock** | **CFU/g** |
| --- | --- | --- | --- | --- | --- | --- |
| Root | U.G.B. | (1.13±3.39) 10^7^ |  | Rhizosphere | U.G.B. | (4.11±1.40) 10^9^ |
|  | SO4 | (1.33±6.79) 10^7^ |  |  | SO4 | (2.76±1.10) 10^9^ |
|  | 420A | (2.48±1.02) 10^6^ |  |  | 420A | (2.63±7.46) 10^9^ |
|  | 157.11 | (4.25±9.69) 10^7^ |  |  | 157.11 | (1.64±2.83) 10^10^ |
|  | 161.49 | (4.43±1.73) 10^7^ |  |  | 161.49 | (5.00±2.79) 10^9^ |

**Additional file Table S19**. Phylogenetic identification of one representative bacterial isolates for each ITS-haplotype. Asterisks (*) indicate the bacterial strains that matched with the main OTUs shared by all the analyzed samples (see Figure 4).

| **Rootstock** | **Fraction** | **ID isolate** | **ITS** | ***** | **Closest described relative (BLST)** | **Acc N.** | **Identity (%)** | **Base** |
| --- | --- | --- | --- | --- | --- | --- | --- | --- |
| 157.11 | Root | 157-E01 | 3 |  | *Serratia proteamaculans* | KX035070 | 100 | 551/551 |
|  | Root | 157-E02 | 20 |  | *Serratia liquefaciens* | KC191827 | 99 | 533/539 |
|  | Root | 157-E07 | 10 |  | *Rahnella aquatilis* | U90757 | 99 | 935/937 |
|  | Root | 157-E09 | 3 | * | *Pseudomonas chlororaphis* | FJ652608 | 100 | 933/933 |
|  | Root | 157-E11 | 12 |  | *Sphingobacterium multivorum* | EU240954 | 97 | 926/935 |
|  | Rhizo | 157-R01 | 13 | * | *Enterobacter amnigenus* | NR_024642 | 99 | 931/933 |
|  | Rhizo | 157-R02 | 3 |  | *Ochrobactrum pseudogrignonense* | GQ203110 | 99 | 907/909 |
|  | Rhizo | 157-R06 | 3 |  | *Serratia liquefaciens* | KC191827 | 99 | 604/605 |
|  | Rhizo | 157-R11 | 1 |  | *Achromobacter xylosoxidans* | AF439314 | 99 | 927/928 |
|  | Rhizo | 157-R13 | 10 | * | *Enterobacter aerogenes* | AB244467 | 99 | 658/659 |
|  | Rhizo | 157-R23 | 1 | * | *Pseudomonas putida* | DQ208660 | 99 | 819/821 |
|  | Rhizo | 157-R24 | 2 | * | *Enterobacter asburiae* | EU221358 | 99 | 925/926 |
|  | Rhizo | 157-R28 | 7 | * | *Enterobacter aerogenes* | FJ976592 | 98 | 725/733 |
|  | Rhizo | 157-R32 | 1 | * | *Enterobacter aerogenes* | AB099402 | 100 | 929/929 |
|  | Rhizo | 157-R37 | 1 |  | *Delftia tsuruhatensis* | DQ356901 | 100 | 925/925 |
|  | Rhizo | 157-R39 | 2 | * | *Pseudomonas putida* | GU073466 | 99 | 922/923 |
|  | Rhizo | 157-R40 | 3 | * | *Pseudomonas rhodesiae* | AF064459 | 100 | 641/641 |
|  | Rhizo | 157-R43 | 3 | * | *Enterobacter cloacae* | GU549440 | 99 | 926/928 |
| 161.49 | Root | 161-E07 | 19 | * | *Pseudomonas fluorescens* | GU198105 | 99 | 902/912 |
|  | Root | 161-E25 | 5 |  | *Acinetobacter lwoffii* | KP790045 | 99 | 909/915 |
|  | Root | 161-E38 | 12 | * | *Pseudomonas mandelii* | EU586044 | 99 | 620/621 |
|  | Rhizo | 161-R01 | 46 | * | *Enterobacter aerogenes* | GU265554 | 99 | 779/782 |
|  | Rhizo | 161-R28 | 1 | * | *Pseudomonas koreensis* | GU185861 | 99 | 613/614 |
|  | Rhizo | 161-R41 | 1 | * | *Enterobacter aerogenes* | GU265554 | 99 | 776/778 |
| 420A | Root | 420A-E02 | 46 | * | *Enterobacter cloacae* | Y17665 | 99 | 904/907 |
|  | Root | 420A-E05 | 12 | * | *Cronobacter sakazakii* | GU252299 | 99 | 896/899 |
|  | Root | 420A-E11 | 6 | * | *Enterobacter aerogenes* | AB244456 | 99 | 826/827 |
|  | Root | 420A-E13 | 34 |  | *Paenibacillus illinoisensis* | JQ579623 | 99 | 902/907 |
|  | Root | 420A-E22 | 1 |  | *Paenibacillus polymyxa* | KT783525 | 99 | 913/917 |
|  | Root | 420A-E32 | 2 | * | *Enterobacter aerogenes* | CP014748 | 99 | 821/823 |
|  | Root | 420A-E49 | 2 |  | *Paenibacillus polymyxa* | KT783525 | 99 | 912/917 |
|  | Root | 420A-E50 | 5 |  | *Bacillus amyloliquefaciens* | CP018200 | 99 | 775/778 |
|  | Rhizo | 420A-R01a | 30 | * | *Pseudomonas plecoglossicida* | KY511070 | 99 | 854/861 |
|  | Rhizo | 420A-R01b | 31 | * | *Klebsiella oxytoca* | AY601679 | 100 | 932/932 |
|  | Rhizo | 420A-R03 | 7 | * | *Enterobacter amnigenus* | JQ062999 | 100 | 740/740 |
|  | Rhizo | 420A-R13 | 2 | * | *Pseudomonas plecoglossicida* | KY511070 | 99 | 899/905 |
|  | Rhizo | 420A-R19 | 6 | * | *Klebsiella oxytoca* | AY601679 | 99 | 929/930 |
|  | Rhizo | 420A-R28 | 1 |  | *Stenotrophomonas maltophilia* | LT222224 | 99 | 624/626 |
|  | Rhizo | 420A-R29 | 11 |  | *Serratia rubidaea* | AB680354 | 99 | 929/930 |
|  | Rhizo | 420A-R38 | 15 | * | *Klebsiella oxytoca* | AY601679 | 100 | 934/934 |
| SO4 | Root | SO4-E01 | 20 | * | *Pseudomonas chlororaphis* | KU977134 | 100 | 929/929 |
|  | Root | SO4-E16 | 1 | * | *Pseudomonas putida* | KU977120 | 100 | 930/930 |
|  | Root | SO4-E17 | 8 | * | *Enterobacter cloacae* | KY229749 | 100 | 928/928 |
|  | Root | SO4-E26 | 2 | * | *Enterobacter aerogenes* | KU726959 | 100 | 930/930 |
|  | Root | SO4-E36 | 1 | * | *Enterobacter cloacae* | CP017186 | 100 | 932/932 |
|  | Root | SO4-E51 | 23 |  | *Rahnella aquatilis* | JX860521 | 100 | 907/907 |
|  | Rhizo | SO4-R01 | 21 | * | *Enterobacter aerogenes* | CP014748 | 100 | 606/606 |
|  | Rhizo | SO4-R02 | 30 | * | *Enterobacter cloacae* | KY229749 | 100 | 946/946 |
|  | Rhizo | SO4-R03 | 6 | * | *Pseudomonas plecoglossicida* | KY511070 | 100 | 930/930 |
|  | Rhizo | SO4-R06 | 6 | * | *Enterobacter ludwigii* | CP017280 | 100 | 895/895 |
|  | Rhizo | SO4-R08 | 1 | * | *Pantoea agglomerans* | LN997968 | 99 | 568/575 |
|  | Rhizo | SO4-R09 | 3 | * | *Pseudomonas chlororaphis* | LT629747 | 99 | 650/651 |
|  | Rhizo | SO4-R11 | 2 |  | *Sphingobacterium multivorum* | LN995710 | 98 | 642/652 |
|  | Rhizo | SO4-R16 | 2 | * | *Citrobacter freundii* | KM108561 | 99 | 653/659 |
|  | Rhizo | SO4-R19 | 17 | * | *Enterobacter aerogenes* | CP011574 | 99 | 925/926 |
|  | Rhizo | SO4-R37 | 1 | * | *Klebsiella trevisanii* | AF129444 | 99 | 819/822 |
|  | Rhizo | SO4-R38 | 1 | * | *Klebsiella trevisanii* | AF129444 | 99 | 819/822 |
|  | Rhizo | SO4-R43 | 1 |  | *Acinetobacter rhizosphaerae* | LN995703 | 100 | 627/627 |
|  | Rhizo | SO4-R47 | 1 | * | *Pseudomonas plecoglossicida* | KY511070 | 99 | 913/915 |
|  | Rhizo | SO4-R51 | 1 |  | *Sphingobacterium pakistanense* | NR113311 | 98 | 910/929 |
|  | Rhizo | SO4-R53 | 1 | * | *Raoultella planticola* | NR024996 | 100 | 931/931 |
| U.G.B. | Root | BFDP-E01 | 23 | * | *Pseudomonas fluorescens* | AB680976 | 99 | 898/899 |
|  | Root | BFDP-E04 | 22 | * | *Pantoea dispersa* | HF585407 | 99 | 656/659 |
|  | Root | BFDP-E12 | 1 | * | *Pseudomonas mandelii* | KU921534 | 99 | 659/660 |
|  | Root | BFDP-E25 | 5 |  | *Bacillus subtilis* | DQ112340 | 99 | 932/940 |
|  | Rhizo | BFDP-R01 | 26 | * | *Enterobacter cloacae* | CP017184 | 99 | 1011/1012 |
|  | Rhizo | BFDP-R14 | 16 | * | *Citrobacter freundii* | CP018810 | 99 | 855/863 |
|  | Rhizo | BFDP-R22 | 1 |  | *Serratia ficaria* | NR041979 | 100 | 1070/1070 |
| Total |  |  | 636 |  |  |  |  |  |

**Additional file Table S20.** Phylogenetic identification of bacteria isolates from **(A)** root and **(B)** rhizosphere of four rootstocks and one ungrafted Barbera (U.G.B.). The bacterial isolates have been obtained from the three plants sampled for each rootstock. The number reported in the graph indicated the total of bacterial isolates assigned to each bacterial taxon (genus). The total number of bacteria isolated from each fraction/rootstocks was reported in the last row of each table.

| **Root** |  | **157** | **161** | **420A** | **SO4** | **U.G.B** |
| --- | --- | --- | --- | --- | --- | --- |
| Gammaproteobacteria | Acinetobacter |  | 5 |  |  |  |
|  | Cronobacter |  |  | 12 |  |  |
|  | Enterobacter |  |  | 54 | 11 |  |
|  | Pantoea |  |  |  |  | 22 |
|  | Pseudomonas | 3 | 31 |  | 21 | 24 |
|  | Rahnella | 10 |  |  | 23 |  |
|  | Serratia | 23 |  |  |  |  |
| Bacteroidetes | Sphingobacterium | 12 |  |  |  |  |
| Bacilli | Bacillus |  |  | 5 |  | 5 |
|  | Paenibacillus |  |  | 37 |  |  |
| Total |  | 48 | 36 | 108 | 55 | 51 |
|  |  |  |  |  |  |  |
| **Rhizosphere** |  | **157** | **161** | **420A** | **SO4** | **BFDP** |
| Alphaproteobacteria | Ochrobactrum | 3 |  |  |  |  |
| Betaproteobacteria | Achromobacter | 1 |  |  |  |  |
|  | Delftia | 1 |  |  |  |  |
| Gammaproteobacteria | Acinetobacter |  |  |  | 1 |  |
|  | Citrobacter |  |  |  | 2 | 16 |
|  | Enterobacter | 36 | 47 | 7 | 74 | 26 |
|  | Klebsiella |  |  | 52 | 2 |  |
|  | Pantoea |  |  |  | 1 |  |
|  | Pseudomonas | 6 | 1 | 32 | 10 |  |
|  | Raoultella |  |  |  | 1 |  |
|  | Serratia | 3 |  | 11 |  | 1 |
|  | Stenotrophomonas | |  | 1 |  |  |
| Bacteroidetes | Sphingobacterium |  |  |  | 3 |  |
| Total |  | 50 | 48 | 103 | 94 | 43 |

**Additional file Table S21.** A bacterial strain representative of each haplotype was assayed for inorganic phosphate solubilisation, siderophore release and auxin synthesis. The data were presented on the base of i) fraction of origin (root or rhizosphere), and ii) rootstock type (ungrafted U.G.B and grafted 157, 161, SO4 and 420A).

| **Rootstock** | **Fractions** | **N° of isolates** | **N° (%) of isolates with PGP traits^†^** | | | |
| --- | --- | --- | --- | --- | --- | --- |
|  |  |  | **ACCd** | **P. solub.** | **Siderop.** | **IAA** |
| U.G.B. | Root | 4 | 4 (100) | 3 (75) | 3 (75) | 4 (100) |
|  | Rhizosphere | 3 | 3 (100) | 3 (100) | 3 (100) | 3 (100) |
| SO4 | Root | 6 | 6 (100) | 4 (70) | 4 (70) | 5 (80) |
|  | Rhizosphere | 15 | 15 (100) | 7 (45) | 6 (40) | 11 (75) |
| 420A | Root | 8 | 8 (100) | 4 (50) | 0 (0) | 8 (100) |
|  | Rhizosphere | 8 | 8 (100) | 6 (75) | 6 (75) | 8 (100) |
| 157.11 | Root | 5 | 5 (100) | 3 (60) | 3 (60) | 3 (60) |
|  | Rhizosphere | 13 | 13 (100) | 10 (80) | 8 (60) | 12 (90) |
| 161.49 | Root | 3 | 3 (100) | 0 (0) | 1 (35) | 2 (70) |
|  | Rhizosphere | 3 | 3 (100) | 2 (67) | 2 (67) | 2 (67) |
| **Grapevine fraction** | | **N° of isolates** | **% of isolates with PGP traits^†^** | | | |
|  |  |  | **ACCd** | **P. solub** | **Siderof.** | **IAA** |
| Root | | 26 | 100 | 54 | 42 | 85 |
| Rhizosphere | | 42 | 100 | 67 | 60 | 86 |
| Total collection of isolates | | 68 | 100 | 62 | 53 | 85 |

^†^PGP traits: Plant growth promoting traits; ACCd = 1-aminocyclopropane-1-carboxylate deaminase activity; P. solub.= phosphate solubilization; Siderop.= siderophore production; IAA = Indole acetic acid production.

**ADDITIONAL FILE FIGURES**

**Additional file Figure S1. Sampling scheme.** Root and rhizospheric soil were collected from ungrafted and grafted *Vitis vinifera* L. var *Barbera* adult grapevine plants growing in a vineyard of the ‘Le Fracce’ farm (Oltrepò Pavese, Italy). Among the grafted grape plants, four rootstocks *Vitis berlandieri* × *Vitis riparia* were sampled: SO4, 420A, 161.49 and 157.11. Three replicates per sample were collected. Unvegetated bulk soil samples (n=3) have been also sampled as control reference.


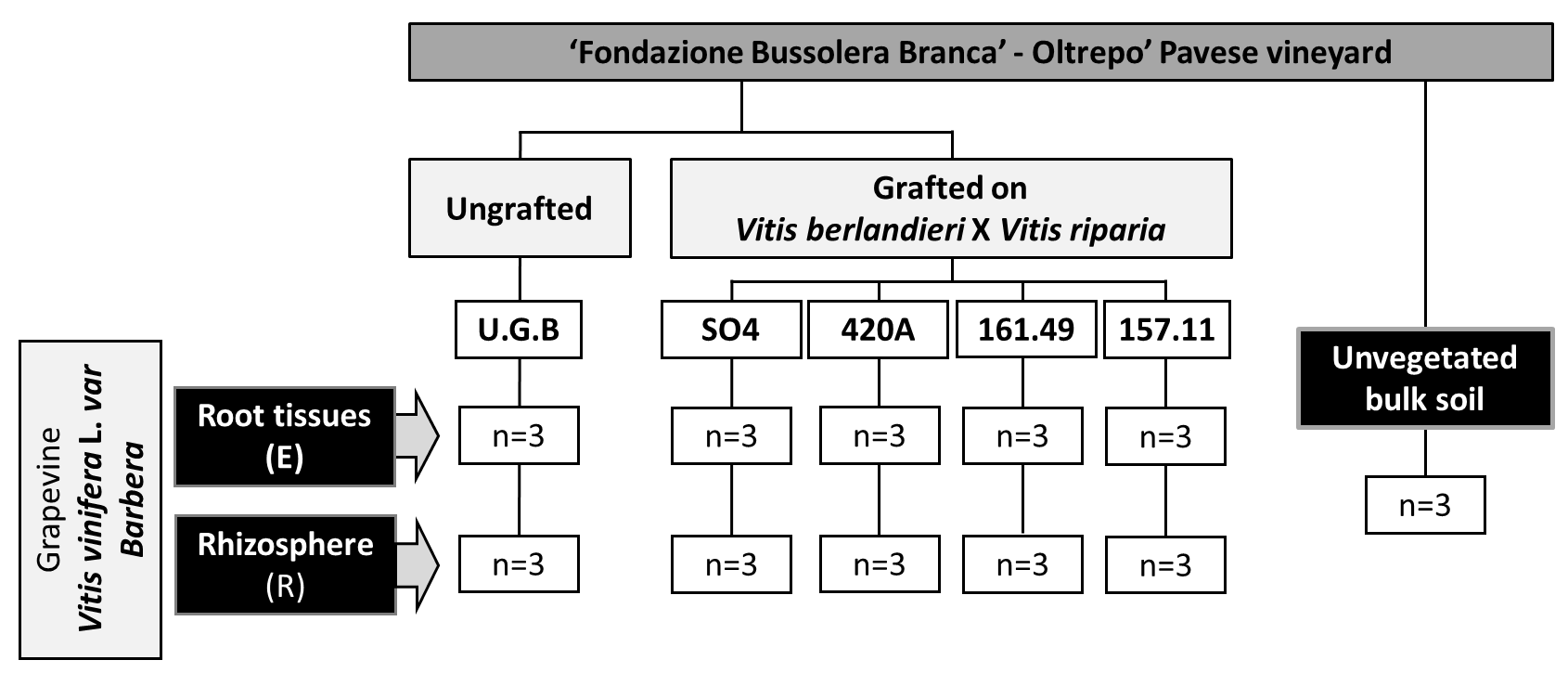


**Additional file Figure S2. Phylogenetic diversity and functional prediction of bacterial community associated to root tissues and rhizosphere of grafted and ungrafted Barbera plants.** Principal Coordinate Analysis (PCoA) showed the rootstock effect on (**A** and **B**) beta-diversity, (**C** and **D**) on the metabolism prediction, and (**E** and **F**) PGP trait prediction associated to root and rhizosphere fractions, respectively. Diamonds: endophytic bacterial community; Circle: rhizospheric bacterial community; Blue: Ungrafted Barbera; Light grey: 161.49; Dark grey: SO4; White: 157.11; Black: 420A.


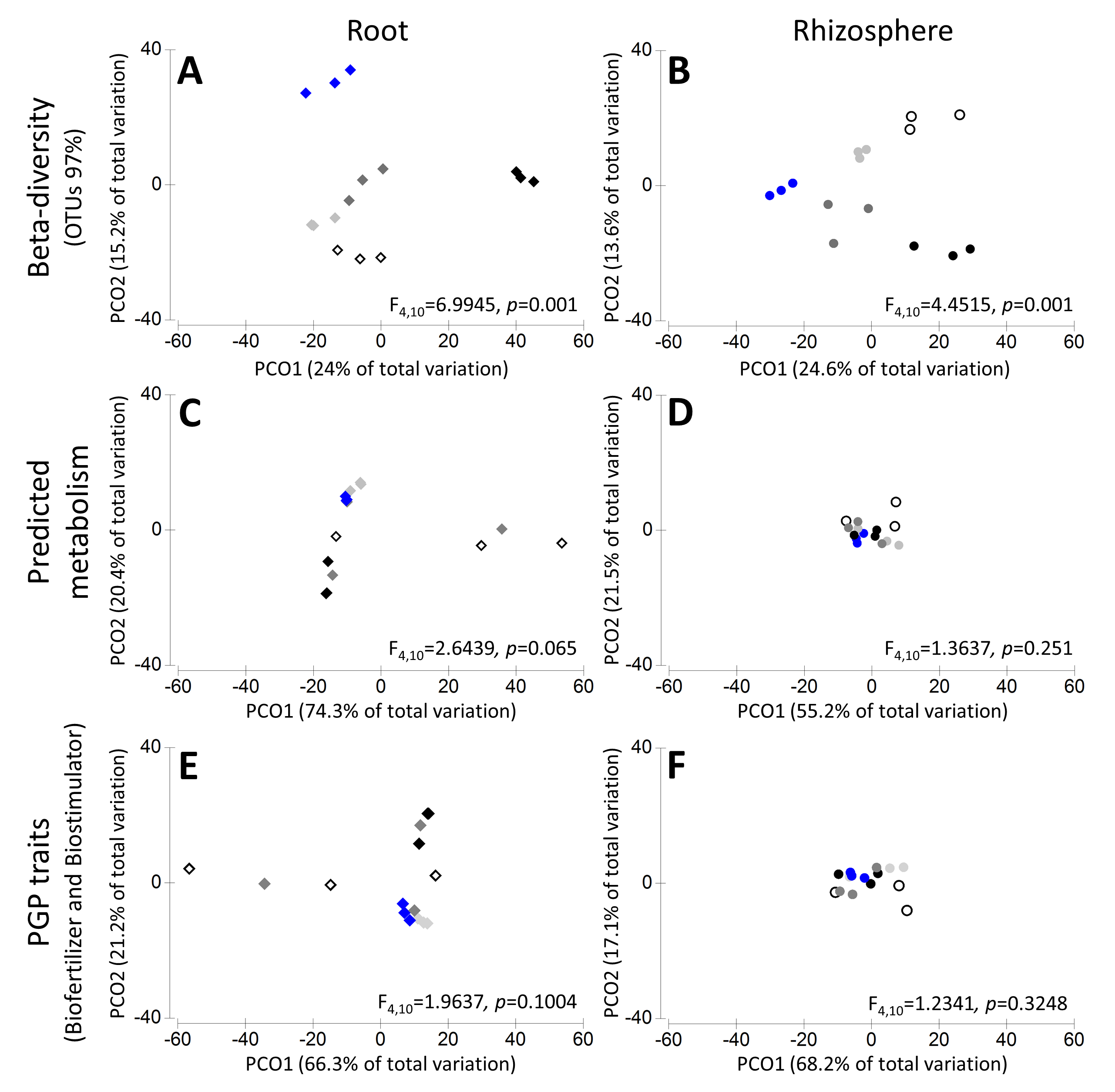


**Additional file Figure S3.** Alpha-diversity expressed as **(A)** total number of OTUs and **(B)** Shannon index in the root and rhizosphere of ungrafted (U.G.B) and grafted (157,161, 420A and SO4) grape plants.

**
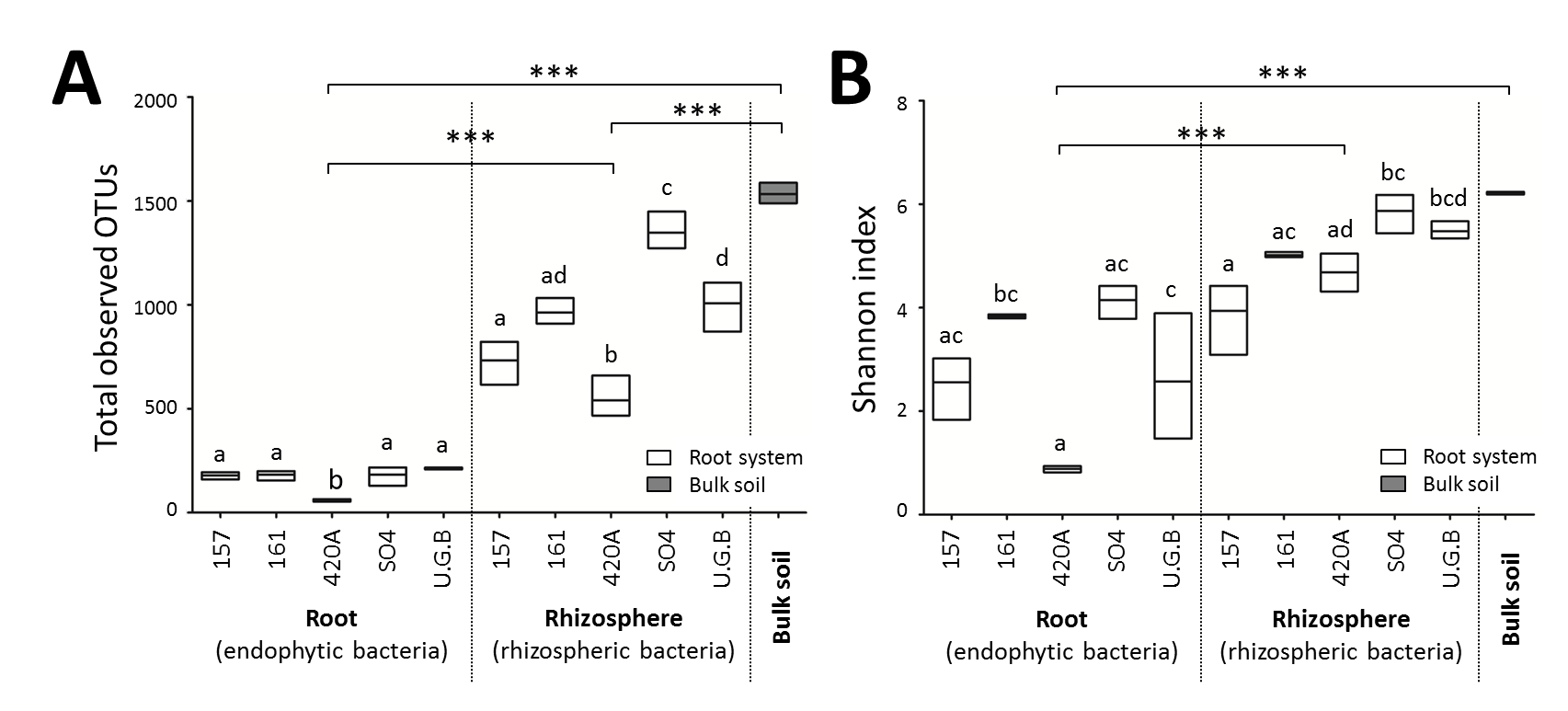
**

**Additional file Figure S4.** Relative abundance of bacterial genera belonging to *Actinobacteria*, *Alphaproteobacteria* and *Gammaproteobacteria.*

**
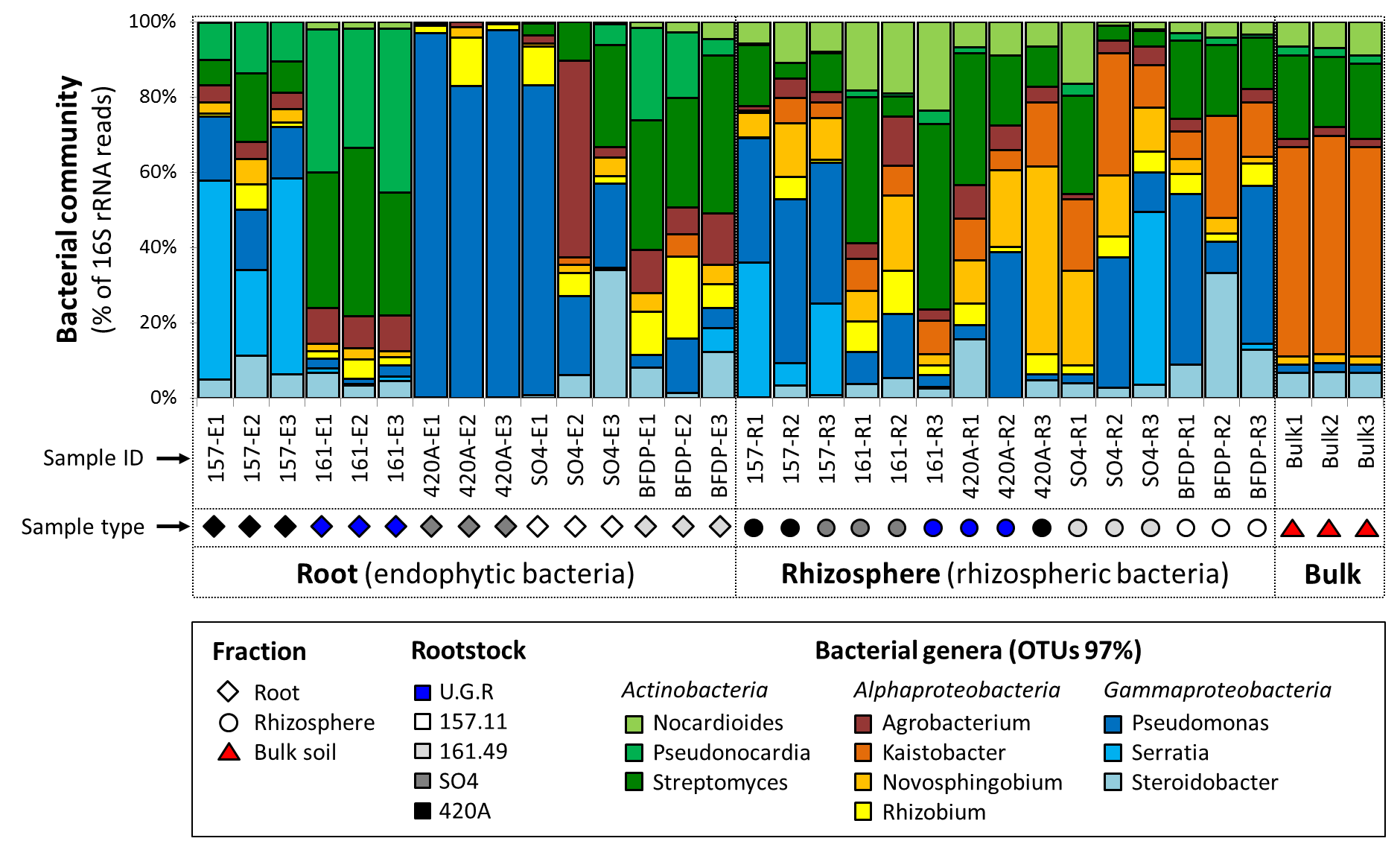
**

**Additional file Figure S5.** Box plot of nodes degree distribution of co-occurrence network analysis for grafted and ungrafted grape root system (GLM, df=1,4, Deviance= 24189, *p*<0.0001).

**Additional file Figure S6.** Node degree distribution in grafted and ungrafted grape root system network analysis. Only node in the third percentile have been consider (See Supporting Figure S6 and Additional file Table S13)

**
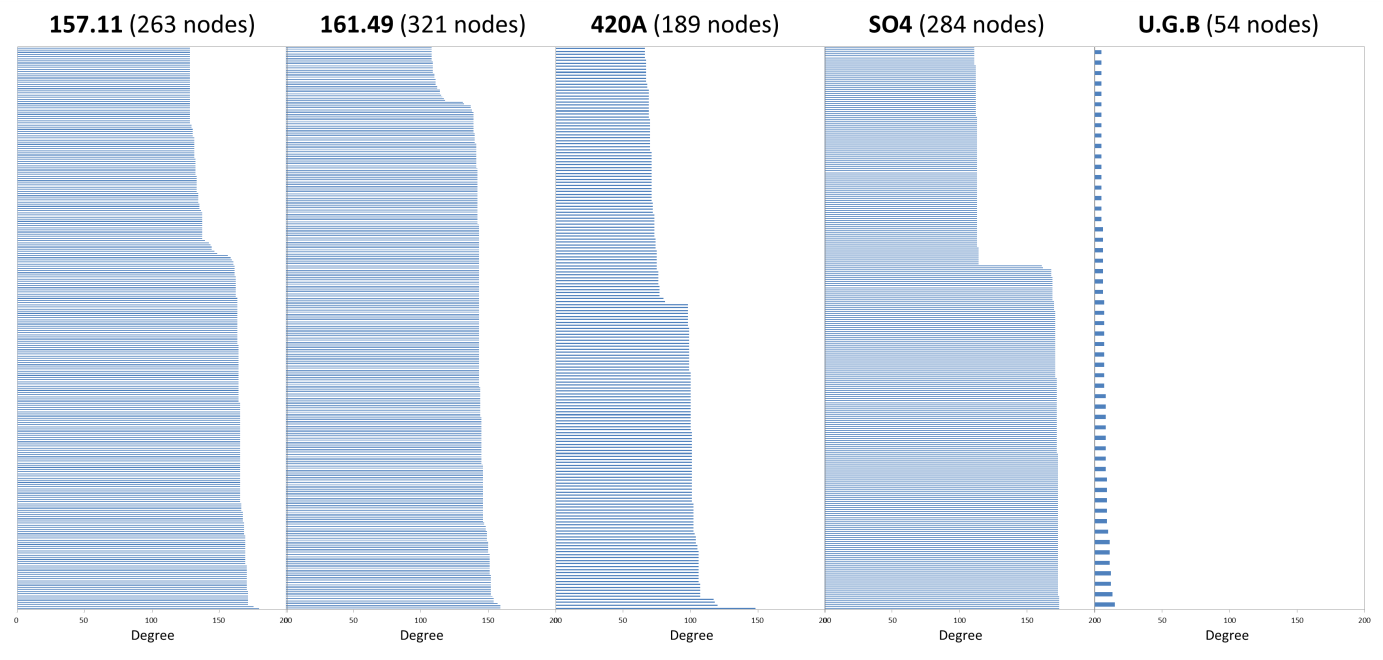
**

**Additional file Figure S7.** Phylogenetic tree of ‘*Candidatus* Phytoplasma’ OTU6

**Additional file Figure S8. (A)** Relative abundances of PGP traits in root and rhizosphere of grape grafted and ungrafted bacterial community. Key genes for Auxin production, nitrogen metabolism, phosphate solubilization, siderophore synthesis, ACC deaminase activity and general PGP activity such as VOCs production were combined. Their mean abundance in root tissues was plotted against the mean abundance in rhizospheric soils, considering all the different plants together. Size and colour of the circles indicate the number of genes involved in the different metabolisms. The enzymes included in the analysis are given in Additional file Table S8. **(B)** Heatmap based on mean abundances of gene with KEGG orthologs involved in PGP traits. The mean relative abundance is showed in root (E) and rhizosphere (R) fractions for ungrafted (U.G.B) and grafted (157.11, 161.49, 420A and SO4) grape. Light: low relative abundance; Light green: mean relative abundance; Dark green: high relative abundance.

**
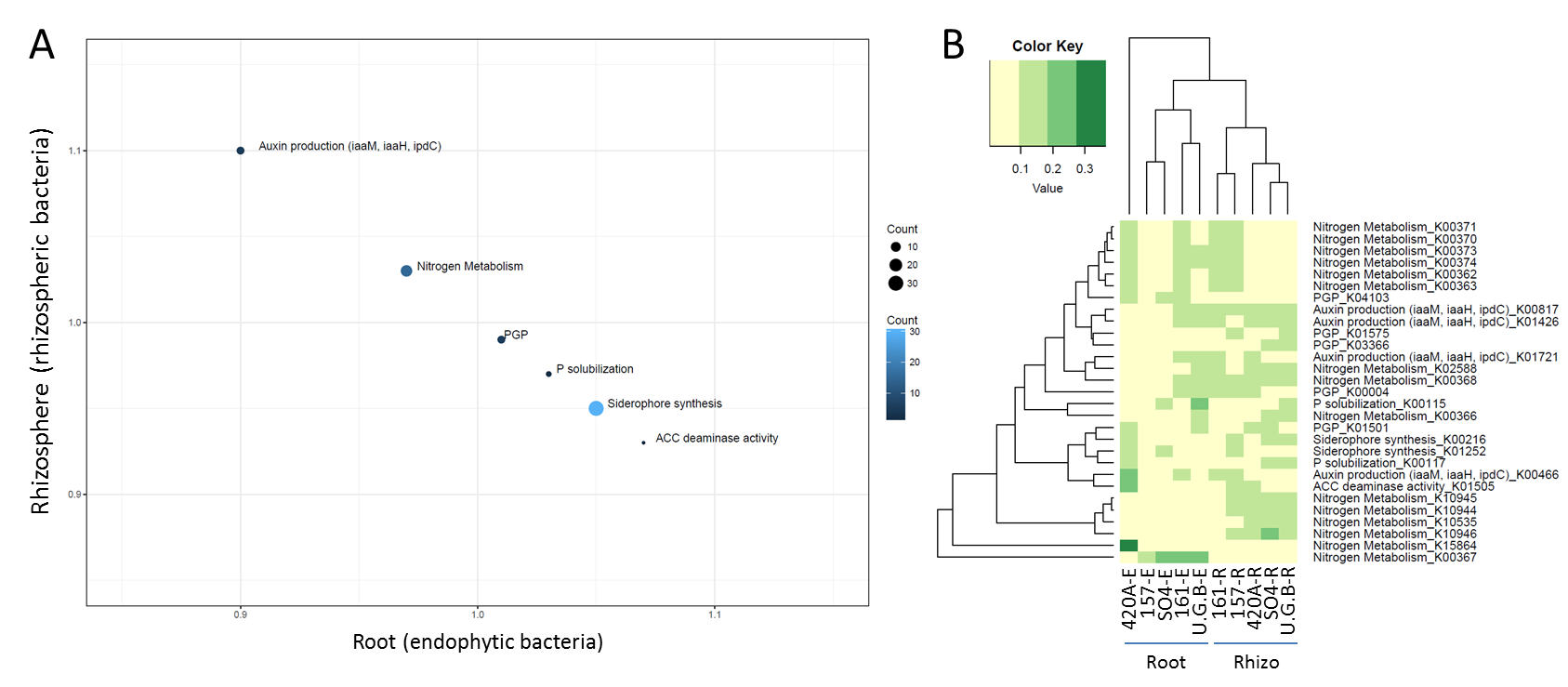
**

**Additional file Figure S9.** Rarefaction curve and Good’s coverage values have been calculated for each sample.


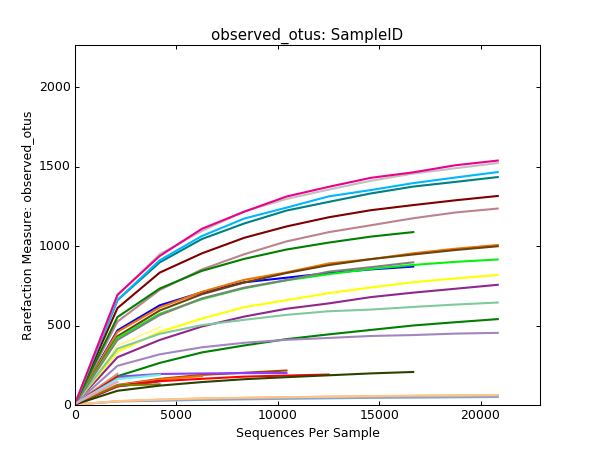


| **Root samples** | **Good's coverage** |  | **Rhizosphere samples** | **Good's coverage** |  | **Bulk samples** | **Good's coverage** |
| --- | --- | --- | --- | --- | --- | --- | --- |
| 157E1 | 0.998 |  | 157R1 | 0.994 |  | B1 | 0.986 |
| 157E2 | 0.991 |  | 157R2 | 0.989 |  | B2 | 0.990 |
| 157E3 | 0.992 |  | 157R3 | 0.990 |  | B3 | 0.988 |
| 161E1 | 0.994 |  | 161R1 | 0.992 |  |  |  |
| 161E2 | 0.996 |  | 161R2 | 0.988 |  |  |  |
| 161E3 | 0.995 |  | 161R3 | 0.994 |  |  |  |
| 420AE1 | 0.999 |  | 420AR1 | 0.996 |  |  |  |
| 420AE2 | 0.999 |  | 420AR2 | 0.999 |  |  |  |
| 420AE3 | 0.999 |  | 420AR3 | 0.960 |  |  |  |
| U.G.B.E1 | 0.985 |  | U.G.B.R1 | 0.989 |  |  |  |
| U.G.B.E2 | 0.999 |  | U.G.B.R2 | 0.988 |  |  |  |
| U.G.B.E3 | 1.000 |  | U.G.B.R3 | 0.989 |  |  |  |
| SO4E1 | 0.996 |  | SO4R1 | 0.993 |  |  |  |
| SO4E2 | 0.995 |  | SO4R2 | 0.990 |  |  |  |
| SO4E3 | 0.985 |  | SO4R3 | 0.989 |  |  |  |
